# Autoimmune Heart Disease: A Comprehensive Summary for Forensic Practice

**DOI:** 10.3390/medicina59081364

**Published:** 2023-07-25

**Authors:** Eleonora Mezzetti, Andrea Costantino, Matteo Leoni, Rebecca Pieretti, Marco Di Paolo, Paola Frati, Aniello Maiese, Vittorio Fineschi

**Affiliations:** 1 Department of Surgical, Medical and Molecular Pathology and Critical Care Medicine, Institute of Legal Medicine, University of Pisa, 56126 Pisa, Italy; eleonora.mezzetti@gmail.com (E.M.); andrea.costantino.med@gmail.com (A.C.); matteo.leoni.pisa@gmail.com (M.L.); rebeccapieretti@gmail.com (R.P.); marco.dipaolo@unipi.it (M.D.P.); 2 Department of Anatomical, Histological, Forensic and Orthopedical Sciences, Sapienza University of Rome, Viale Regina Elena 336, 00161 Rome, Italy; paola.frati@uniroma1.it (P.F.); vittorio.fineschi@uniroma1.it (V.F.)

**Keywords:** cardiomyopathy, autoimmune pathology, cardiac involvement

## Abstract

Autoimmune heart disease is a non-random condition characterised by immune system-mediated aggression against cardiac tissue. Cardiac changes often exhibit nonspecific features and, if unrecognised, can result in fatal outcomes even among seemingly healthy young individuals. In the absence of reliable medical history, the primary challenge lies in differentiating between the various cardiopathies. Numerous immunohistochemical and genetic studies have endeavoured to characterise distinct types of cardiopathies, facilitating their differentiation during autopsy examinations. However, the presence of a standardised protocol that forensic pathologists can employ to guide their investigations would be beneficial. Hence, this summary aims to present the spectrum of autoimmune cardiopathies, including emerging insights such as SARS-CoV-2-induced cardiopathies, and proposes the utilisation of practical tools, such as blood markers, to aid forensic pathologists in their routine practice.

## 1. Introduction

Autoimmune diseases (ADs) are inflammatory syndromes that commonly involve multiple structures and organs. Cardiac involvement is prevalent and associated with elevated cardiovascular morbidity and mortality rates.

Over the past few decades, the prevalence of ADs has markedly increased due to improved detection and surveillance, affecting approximately 7.6–9.4% of the global population. The frequency of cardiac manifestations in various systemic autoimmune diseases remains uncertain and varies depending on the diagnostic methods employed and patient selection. Advancements in imaging technologies and the greater accessibility of diagnostic imaging have led to the identification of a higher frequency of cardiac abnormalities in patients with systemic autoimmune diseases compared to previous autopsy studies [1,2,3,4].

ADs may be due to several structural changes to heart tissue, resulting in different manifestations depending on the cardiac structure involved:

– Endocardium: endocarditis, valvular diseases, thrombi;
– Myocardium: myocarditis, cardiomyopathy, rhythm and conduction disturbances, heart failure;
– Pericardium: pericarditis, pericardial effusion.
– Coronary arteries: acute coronary syndrome, ischemic heart disease, vasculitis

The heart can be directly affected by autoimmune diseases, resulting in damage to its structures, or it can be indirectly involved through chronic inflammation, damage to other organs or the use of medications for managing the underlying disease. The mechanisms underlying the pathological involvement of the heart are not fully understood but involve various processes. In cases of primary cardiac involvement, the impairment of cardiac structures is attributed to an inflammatory infiltrate, deposition of immune complexes and subsequent activation of the complement system. Chronic inflammation, on the other hand, leads to accelerated atherosclerotic processes and upregulation of endothelial adhesion molecules, promoting a prothrombotic environment. Furthermore, persistent inflammation induces oxidative stress and increases fibroblast activity, resulting in the deposition of collagen and interstitial fibrosis in the myocardium [5].

Early clinical manifestations of autoimmune heart damage are often insidious or non-specific and, when symptoms are manifest, the heart damage is often severe and irreversible [6].

Sometimes the existence of an autoimmune disease with cardiac involvement can also lead to sudden cardiac death in young subjects, as in Kawasaki disease [7]. Therefore, early recognition and management of traditional cardiovascular risk factors is essential, along with aggressive treatment with disease-modifying agents to improve the long-term prognosis of these patients.

Currently, there are 80–100 described cardiac diseases that occur because of autoimmune responses and can be classified as follows (Table 1):

The objective of this article is to compile and organise information regarding cardiac involvement in autoimmune diseases. The purpose is to provide guidance and support to pathologists or coroners in determining the cause of death in individuals with autoimmune diseases, particularly when the heart is suspected to be the underlying cause. By presenting relevant insights and findings, this article aims to aid in the accurate identification and understanding of cardiac-related fatalities in individuals with autoimmune conditions [8].

Indeed, a particular focus will be given to blood markers: they refer to the various molecules that can be detected using advanced technologies in blood samples. By integrating the data obtained from blood markers with autopsy findings, it is possible to facilitate a more expedient and accurate diagnosis. The article will underscore the importance of leveraging blood markers as a complementary diagnostic tool to enhance the speed and reliability of diagnosing autoimmune cardiac conditions.

## 2. Materials and Methods

This paper was carried out according to the Preferred Reporting Items for Systematic Review (PRISMA) standards [9]. Before the individual chapters were written, a scientific literature review of the collected studies was conducted using the electronic search of PubMed, Science Direct Scopus, Google Scholar, and the Excerpta Medica Database (EMBASE). 

The used search terms were “cardiomyopathy”, “cardiopathy”, “autoimmune pathology”, “forensic practice”, “cardiac histopathology”, “immunohistochemistry”, “genetic findings”, “blood markers”, and “autopsy” in the title, abstract, and keywords. The bibliographies of all located papers were examined and cross-referenced to identify relevant literature further. Only the characteristics of cardiac pathology in autoimmune diseases were investigated without delving into the remaining phenotype.

**Figure 1:**
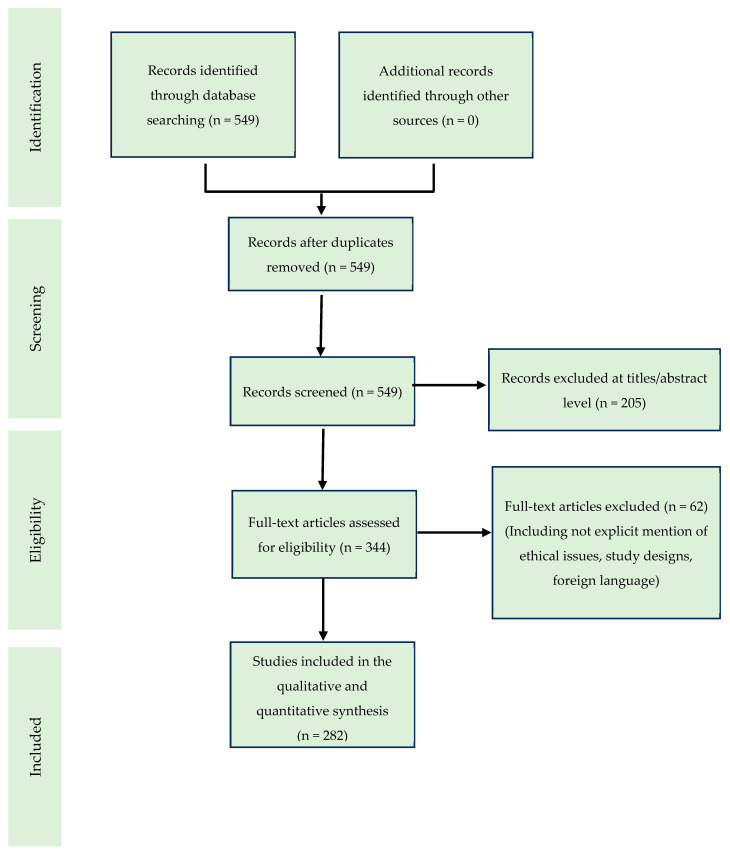
The figure shows the PRISMA Flow Diagram designed according to PRISMA Guidelines.

A methodological appraisal of each study was conducted according to the PRISMA standards, including an evaluation of bias. The data collection process included study selection and extraction (Figure 1). Two researchers (A.M. and E.M.) independently examined the papers with titles or abstracts that appeared to be relevant and selected the papers that described useful findings in autoimmune heart disease. Disagreements concerning eligibility among the researchers were resolved by consensus. Preprint articles were excluded, including only English papers or papers presenting an English version. Data extraction was performed by four investigators (A.C., E.M., M.L., and R.P.), and four other investigators (A.M., M.D.P, P.F., and V.F.) verified the extracted data again. All duplicates, such as all full-text articles, were excluded without explicitly mentioning ethical issues or study design. Case reports, and reviews of previously published literature were also considered. This study was exempt from institutional review board approval. Human subjects were not involved.

## 3. Cardiopathy in Churg-Strauss Syndrome

Churg-Strauss syndrome (CSS) is an inflammatory granulomatous vasculitis affecting medium and small vessels, first described in 1951. It predominantly affects males from 30 to 60 years. 

The main clinical features are described in the below table (Table 2) [9,10,11]. 

### 3.1. Pathogenesis

The occurrence of CSS is closely correlated with blood and tissue hypereosinophilia. Although increased eosinophils are also present in other small vessel vasculitis, their tissue-level excess in a patient with small vessel vasculitis makes the diagnosis of Churg Strauss syndrome particularly likely. 

However, the onset of the disease is secondary to a genetic predisposition to which contact with an antigen is added, causing an excessive Th2 and Th17 response [12,13,14,15].

Specifically, the natural history of CSS can be divided in three main phases: development of asthma, involving Th2 lymphocytes; potential role of eosinophils infiltrating tissues; contribution of Myeloperoxidase antineutrophil cytoplasmic antibodies (MPO ANCA) to the formation of vasculitis lesions.

Genetic investigations have identified a reliable mutation of *HLA-DRB4* and *DNAM1s* genes [16,17]. 

### 3.2. Cardiac Clinical Findings

Cardiac involvement is observed in up to 60% of cases (17–92%) and 48% of fatal forms. In this case, death often occurs within the first few months following diagnosis.

The main cardiac clinical findings are summarised in the following table (Table 3) [18,19].

### 3.3. Cardiac Histology

CSS has a systemic involvement. This pathology may be or not granulomatous and characteristically involves both arteries and veins as well as pulmonary and systemic vessels. Granulomas are typical lesions about a centimetre in diameter, located near small arteries or veins in the lung, gastro-enteric tract, or skin. They are characterised by epithelioid histiocytes arranged in a palisade pattern around a necrotic amorphous granular centre, where eosinophils are predominant [20].

Cutaneous granulomas are not pathognomonic of CSS but can be seen in another vasculitis (granulomatosis with polyangiitis, microscopic polyangiitis) or autoimmune diseases (systemic lupus erythematosus, rheumatoid arthritis).

At hematoxylin-eosin, eosinophilic pericarditis, eosinophilic myocarditis, and eosinophilic or granulomatous coronary arteritis are the predominant cardiac findings. Eosinophilic infiltration can be confirmed by Giemsa staining.

Histologically, myocardial granulomatous infiltration and coronary vasculitis are the most frequent lesions [21]. At the myocardial level, diffuse or spot-accumulated eosinophilic infiltrates, including leukocytes, are frequently evident; in the endocardium, eosinophil cationic protein, degranulated eosinophils, and activated eosinophils can be found, involving the myocardial interstitial too [22,23].

For these characteristics, cardiac involvement appears to overlap with Loeffer’s myocarditis [24]. Endomyocardial fibrosis is a rarer finding in both ventricles, and it can be accompanied by macrophages and lymphocyte infiltration. In severe cases, fibrosis can be strongly represented, assuming an appearance like restrictive cardiomyopathy [25].

As in the rest of the body, small vessels are affected by necrotizing vasculitis, accompanied by neutrophilic perivascular infiltrates and extravascular granulomas. Large mural thrombi might develop in both ventricles, resulting in a reduction of ventricular cavities size. Coronary arteries are affected by vasculitis phenomena that, in some cases, can lead to stenosis or ectasia [26,27].

The study of cardiac tissue can also help in the dating of cardiac disease: in the acute phase, severe eosinophilic endomyocarditis is evident, while in the subacute phase, interstitial fibrosis can be found. As for mural thrombi, they are typical of the chronic phase, approximately ten months after onset [28].

### 3.4. Cardiac Immunohistochemistry and Immunofluorescence

In immunohistochemistry, using labelled antibodies such as EG2 allows the detection of a common epitope of ECP and eosinophil protein-X, markers strongly positive in eosinophils. These markers are mainly represented in the endocardium and, less intensely, in the myocardial interstitial [29]. Similarly, the study with antibodies for eotaxin-3 showed discrete positivity at the level of endothelial cells of small vessels in both arterioles and venules. Eotaxin-3 expression was also highlighted in smooth muscle cells of small arterioles. As a result, eotaxin-3 expression is detectable at sites of active disease and accompanied by Th2 lymphocyte infiltration [30].

At immunofluorescence, Nakayama et al. detected CD3-positive lymphocytes and CD68-positive macrophages infiltrating myocardial tissue [31].

Schoppet et al. confirmed these findings by showing the presence of a diffuse CD68+HLA-DR+ dendritic cell (DCs) in the myocardium with a reduction of CD56 expression (neural cell adhesion molecule) [32].

In at least 40% of cases, ANCA perinuclear immunofluorescent pattern is present with specificity for myeloperoxidase (MPO) [33]. However, in some studies, negativity for the ANCA pattern was associated with a higher prevalence of cardiomyopathy and less risk of vasculitis [34].

### 3.5. Genetic and Blood Markers

Different markers can be found at the blood level and in the active phase. Indeed, the main typical findings of CSS are blood hyper-eosinophilia, and high IgE levels (75%) [35].

CSS is strongly associated with ANCA [36], especially anti-MPO; in the case of cardiac involvement ANCA positivity occurs in 40% of cases, while even in cases of active disease it is much more common to be negative (19–33%). High serum IgG levels have been reported in some cases of cardiac involvement with undetermined ANCA levels [37]. Moreover, serum CCL17 and IgG4 levels relate with disease activity [38]. Some patients also tested positive for rheumatoid factor [39].

Cardiac necrosis markers such as Troponin I and Creatin-kinase will be elevated in case of myocarditis or ischemic pathology. Numerous studies have also shown elevated levels of pro-inflammatory cytokines, specifically IL-4, IL-13, IL-5, INF-g, and INF-a [40]. In the end, Poltzer et al. showed that high levels of Eotaxin 1–2 and 3 can also be a strong indicator in CSS [41], while Dallas et al. study revealed significant serum CCL17/TARC levels in patients with active disease [42].

## 4. Cardiopathy in Takayasu Disease

Takayasu’s arteritis (TA) is a rare vasculitis that mostly involves the great vessels. It mostly affects women in their thirties. Recent epidemiologic studies suggest a prevalence within the European population, while the prevalence of TA in Scandinavian countries has increased because of the higher rates of immigration from Asia and Africa. In summary, recent surveys show an increase in prevalence among all ethnic groups.

If cardiac and, specifically, coronary artery involvement occurs, the prognosis is poor. In this pathology, immune inflammation is a typical feature of Takayasu’s disease; the interactions between dendritic cells and lymphocytes may be important in the control of the immune reactions. Complications are more common in men and patients with late referrals [43,44,45].

### 4.1. Pathogenesis

The cellular and biochemical processes involved in the pathogenesis of TA are still being studied. At present, the existence of both cellular and antibody-mediated autoimmune mechanisms is described.

### 4.2. Cardiac Clinical Findings

In TA cardiac involvement is not uncommon. The main cardiac clinical features are summarised in Table 4 [46,47,48,49].

### 4.3. Cardiac Histology

The main histologic changes in cardiac tissue are lymphocytic infiltrates, myocardial hypertrophy and diffuse myocytolysis, as in acute myocarditis. As for coronary artery changes, however, changes in a vasculitic sense are usually present: the main feature is the presence of granulomatous inflammation at the level of the adventitia and middle tunica of the vessels. Fibrotic phenomena may also set in at the level of blood vessels, resulting in stenosis and occlusion. The lesion involving the muscular tunica may evolve into aneurysmal-type changes.

At the level of the tunica intima, it is possible to show the presence of endothelial proliferation associated with fibrosis of the middle and adventitia tunica. At the level of the three layers, an abundant inflammatory infiltrate with increased atherosclerotic phenomena can be observed. Inflammatory nodules of T cells and B cells are not always evident. Dendritic cells are localised with lymphocytes in areas of accumulation of inflammatory infiltrates, while giant cells are not present in the arterial wall affected by Takayasu’s disease [50,51].

### 4.4. Cardiac Immunohistochemistry and Immunofluorescence

In adventitial vasa vasorum, CD3+, CD20+, and S-100+ cells can be found and diffusely distributed around the structure. Some CD15+ cells (granulocytes) are present in the peripheral parts of the inflammatory nodules.

Cardiac myocytes are positive for HLA classes I and II, and ICAM-1, demonstrating the involvement of an active inflammatory process [52,53].

### 4.5. Genetic and Blood Markers

One genetic susceptibility locus identified to date is the HLA-B*52 allele, which has been confirmed in several ethnicities. In addition, other genetic associations have been found with genes encoding regulators of immune response, pro-inflammatory cytokines, and mediators of humoral immunity. Non-HLA susceptibility loci that have recently been identified for *TAK* include *FCGR2A*/*FCGR3A*, *IL12B*, *IL6*, *RPS9*/*LILRB3*, and a locus on chromosome 21 near *PSMG1*. Recently, mutation in the subgroups of B5, *Bw51* and *Bw52* has also been observed in individuals with *TAK*.

Patients with Takayasu arteritis can present an increase in inflammatory markers, such as C-reactive protein and erythrocyte sedimentation rate. In general, systemic inflammatory is not proportional with inflammatory activity in the vessel wall. In some subjects, the so-called “cytokine storm” may occur after a treatment interruption, with increases in IL-1β, IL-1ra, IL-2, IL-4, IL-5, IL-6, IL-8, IL-9, IL-10, IL12p40, IL-12p70, IL-13, IL-15, IL-17, IL-18 IFN-γ, TNF-α); 6 chemokines (Eotaxin, IL-8, IP-10, MCP-1, MIP-1α, MIP-1β), and 4 growth factors (IL-7, G-CSF, GM-CSF, VEGF).

Pentraxin 3 and TNF-α, produced by dendritic cells, vascular smooth muscle cells, fibroblasts, and macrophages, are often elevated. Finally, elevated NT-proBNP levels can be found [54,55,56].

## 5. Cardiopathy in Polyarteritis Nodosa

Polyarteritis Nodosa (PAN) is a rare systemic necrotizing vasculopathy affecting small and medium vessels, first described in 1854. The peak incidence occurs in women between their forties and fifties.

Small arteries are involved but are usually spared. 

The main clinical features are reported in the following table (Table 5) [57]. 

### 5.1. Pathogenesis

The etiology of polyarteritis nodosa is unknown, but immunologic mechanisms appear to be involved. The variety of clinical and anatomopathological features suggests a multifactorial pathogenesis. Usually, no predisposing antigen is detected. 

Most cases are idiopathic, while about 20% of patients have the hepatitis B or C viral infection or hairy cell leukaemia [58]. Some studies show a mutation of *MEFV* gene, but not in all patients. 

### 5.2. Cardiac Clinical Findings

PAN rarely manifests as congestive heart failure, mainly because of extensive coronary artery compromise. Sometimes PAN can result in sudden cardiac death of unknown cause, without coronary involvement. In this case, some authors have proposed that coronary spasm indicates the presence of vasospastic abnormalities in the peripheral circulation, as a sort of Raynaud’s phenomenon.

The involved cardiac structure and clinical manifestations are summarised in Table 6 [59,60,61,62,63,64,65,66,67].

### 5.3. Cardiac Histology

Endomyocardial histology shows active lymphocytic myocarditis associated with small intramural vessels necrotizing vasculitis [68].

Coronary cardiac tissue shows inflammatory changes, with gauge reduction, lymphocytic infiltration of the media, and adventitia, perivascular fibrosis. In some cases, intravascular thrombosis with total or partial occlusion may be evident with or without recanalization. Severe lesions had necrosis and infiltration of the filled thickness of the vessel wall with necrosis and fibrosis of the surrounding perivascular connective tissue. The original components of the arteries can be destroyed and replaced by a moderate or extensive replacement of fibrous tissue.

Atherosclerosis may be a non-collateral finding promoted by inflammatory changes [69]. Small intramyocardial vessels can show extensive lymphocytic infiltration associated with necrosis and lumen obliteration [70].

Focal perivascular and interstitial myocarditis can be observed. In some cases, infiltration of polymorphonuclear leukocytes is shown, suggesting an over-infection.

In infants, valvulitis of the mitral and aortic valves can be found, like rheumatic fever findings. In valves, perivascular inflammation consisting of a central area of fibrinoid necrosis surrounded by inflammatory cells can be documented [71], evolving subsequently in a fibrotic scar.

### 5.4. Cardiac Immunohistochemistry and Immunofluorescence

At the immunohistochemical level, the presence of mostly lymphocytes and macrophages has been documented. Positivity for CD3, CD4, CD8, and CD22 documents the prevalence of lymphocytes at the cardiac level, while granulocytes appear scarce [72].

Positivity for S100 protein reactivity (using avidin-biotin-peroxidase protein), TLR-4, and IL-2R were also observed. On fibroblasts around the vasculitis, bFGF can also be detected [73] such as some of the infiltrating cells around vasculitis lesions expressed VEGF.

At immunofluorescence, in myocarditis, Anti-heart aabs (AHA) and anti-intercalated disk aabs (AIDA) are detectable [74].

### 5.5. Genetic and Blood Markers

In cardiac involvement, increased indices of inflammation, including erythrocyte sedimentation rate, c-reactive protein, and lymphocyte amount are common in the blood. Troponin may be at normal levels and elevate later. Cases of sudden death have been described in cases of concurrent infection with staphylococcus A. In these cases, the anti-streptolysin (ASO) titer can be very high. ANCA, ANA, and rheumatoid factors are always negative [75]. From the genetic point of view, no precise correlations have been observed. Some studies have demonstrated *MEFV* factor and *DADA2* (adenosine diaminase-2) gene expression [76].

## 6. Cardiopathy in Behçet’s Disease

Behçet’s disease (BD) is classified among inflammatory vascular diseases affecting vessels of all kinds and sizes [77,78,79,80]. It is particularly prevalent in the countries along the ancient “Silk Route” populations (e.g., Turkey and Iran); however, the global pool prevalence has been estimated at around 10.3 (95% CI: 6.1–7.7)/100,000 people.

Young adults between the ages of 20 and 40 years are most affected, with BD being more common and more severe in males compared to females [81,82,83,84,85,86]. 

BD can involve the skin, mucosa, joints, eyes, vessels, and nervous and gastrointestinal systems, and so is referred to as a syndrome rather than as a unique and nosologically distinct condition [87,88,89,90,91]. Although the disease rates and the clinical expression vary to some extent by ethnic origin, recurrent mucocutaneous lesions, skin lesions, ocular findings, and reactivity of the skin to needle prick or Injection (pathergy test) constitute common clinical hallmarks of BD [92,93,94,95,96].

As there is a lack of universally recognized pathognomonic tests, BD diagnosis is primarily based on clinical criteria. In 2014, an international team for the revision of the international criteria for BD (ITR-ICBD) was developed from the collaboration of experts from 27 nations to take up the diagnostic quandary and the shortcomings of the preceding criteria, namely the international study group (ISG). The team created a new set of criteria, with the goal of identifying a scheme that has good discriminatory properties regardless of country and that would be intuitive and easy to use in a wide variety of settings (Table 7) [97].

### 6.1. Pathogenesis

BD can be described as a multifactorial disease with an incompletely known etiopathogenesis, and unique geographic distribution suggests that both genetic and environmental susceptibility factors might be involved. It is known that the complex etiopathogenesis of BS involves more than one pathogenetic pathway, namely:

– Genetic and epigenetic factors, including geographic distribution, the association with *HLA* and *non-HLA* genes, and micro-RNA (miRNA) polymorphisms [98,99].

The HLA class I antigen, HLA-B*51, has been identified as the predominant genetic susceptibility factor underlying BD in many populations, proposing that unknown environmental factors may interact with HLA-B*51 alleles.

– Environmental etiology (infections, microbiome, and additional triggering factors) [100];
– Immunological pathways (neutrophils and immune-mediated damage) [101].

### 6.2. Cardiac Clinical Findings

Cardiac involvement has been previously documented in patients with BD. However, its exact incidence, nature, severity, and management need to be better established [102]. The prevalence of cardiac involvement is variable between studies (1–29%), and men seem to be predisposed (male-to-female 14:1) [103,104]. 

The morphological basis of the systemic manifestations in BD, including cardiovascular involvement, is vasculitis [105,106,107,108,109]. It has been reported that venous and arteries of all sizes are affected. It has been reported that venous involvement is 29%, and arterial involvement varies from 8% to 18%. 

The main cardiac structure involved and the clinical picture correlated are summarised in Table 8 [110,111,112,113,114].

### 6.3. Cardiac Histology

BD is classified as a “variable vessel” vasculitides, a term that underlies its atypical histological and clinical features. Indeed, several important clinical and histological differences exist between BD and other systemic vasculitides:

– Contemporary involvement of both arteries and veins of all sizes in BD (venous in general more frequently affected than arteries);
– No clearly reported increased risk of atherosclerosis compared to other vascular inflammatory diseases;
– Unique tendency for aneurysm formation;
– Usual absence of granulomatous inflammatory lesions in the vessel wall, with elastic fibers usually spared [115].

Valvular and vascular involvement is characterized by a coexisting acute and chronic inflammatory process of various stages, distinct from infectious endocarditis, which essentially consists of acute or sub-acute inflammation.

Inflammatory cell infiltrates observed on routine hematoxylin-eosin (HE) stain are confirmed by immunohistochemistry to be acute, with myeloperoxidase (MPO) in neutrophils; they are shown to be chronic with CD3 in T lymphocytes and CD128 in plasma cells.

Unlike the histology of other systemic vasculitides, in BD, these cells are localized more around the vessels than inside the wall. This histologic “perivascular” pattern of BD, probably more like neutrophilic dermatosis than classical systemic vasculitides, has been demonstrated in several districts.

### 6.4. Cardiac Immunohistochemistry and Immunofluorescence

At immunohistochemistry and immunofluorescence, we can find a positivity for MPO (myeloperoxidase), which is a lysosomal neutrophilic enzyme whose implication in tissue damage from Behçet Disease has been demonstrated. CD3 and CD68 are two other main factors detectable at immunohistochemical exam [116].

### 6.5. Genetic and Blood Finding

A wide range of HLA genetic factors has been proposed in association with Behçet Disease, with *HLA-B51* regarded as the most prominent and studied one, especially in the countries of the Silk Road [117]. 

Blood analysis shows an elevation of Anti MPO antibodies in response to myeloperoxidase elevation, while anti-endothelial cell antibodies (AECA), anti-CTD phosphatase subunit-1 (CTDP-1), antinucleon antibodies (ANA), and anti-neutrophil cytoplasm antibodies (ANCA) [118,119].

## 7. Cardiopathy in Kawasaki Disease

Kawasaki disease (KD), or mucocutaneous lymphnode syndrome, is a self-limiting systemic inflammatory disease with a predilection for small and medium size arteries. It predominantly affects young children, mostly under the age of five, with a 1.5 times higher risk in boys than girls [120,121,122,123].

Clinical manifestations of complete KD include prolonged fever (1–2 weeks, mean 10–11 days), conjunctivitis, oral and lip lesions, polymorphous rash, extremity changes, and cervical lymphadenopathy. In addition, an atypical KD includes prolonged fever with atypical clinical manifestations such as arthritis, aseptic meningitis, anterior uveitis, gallbladder hydrops, urethritis, and lung involvement. Some more severely affected patients show cardiac complications (5–20%), particularly coronary artery lesions (CALs) such as aneurysms and ectasias. KD Is the leading cause of acquired heart disease in children living in industrialised countries.

The diagnosis of classic or complete KD utilises clinical criteria. It excludes other similar clinical entities: the patients must have 5 ≥ fever days of fever and ≥4 of the 5 principal clinical features (Table 9). Diagnosing KD is a clinical challenge, given the wide variety of clinical presentations and the similarity to many viral and bacterial illnesses [124]. Therefore, any child with prolonged unexplained fever with any of the principal clinical features should be further evaluated for KD with consideration of echocardiography [125].

### 7.1. Pathogenesis

The leading theory for the pathogenesis of KD is that an unknown infectious agent leads to activation of the immune system in a genetically susceptible child.

To date, no infectious causes have been identified as potential underlying etiologies. The innate immune system may be activated via detection of either pathogen-associated molecular patterns (PAMPs), or damage-associated molecular patterns (DAMPs) [126,127]. In addition, there is also significant activation of the adaptive immune response. There appears to be increased numbers of circulating pro-inflammatory and regulatory T cells in the acute phase of KD [128]. Several auto-antibodies directed against myocardial, endothelial, and extracellular matrix proteins have also been described in the literature, although their clinical significance is poorly understood [129,130].

### 7.2. Cardiac Clinical Findings

Cardiovascular manifestations can be prominent in the acute phase of Kawasaki disease and are the leading cause of long-term morbidity and mortality. The pericardium, myocardium, endocardium, valves, and coronary arteries may be involved during this phase. Cardiac auscultation of the infant or child with KD in the acute phase often reveals a hyperdynamic precordium, tachycardia, a gallop rhythm, and an innocent flow murmur in the setting of anaemia, fever, and depressed myocardial contractility secondary to myocarditis. Children with significant mitral regurgitation may have a pan systolic regurgitant murmur that is typical of this condition. Patients with KD and poor myocardial function may occasionally present with low cardiac output syndrome or shock. Electrocardiography may show arrhythmia, prolonged PR interval, or nonspecific ST and T wave changes. 

The significant sequelae of KD are related to the cardiovascular and, more specifically, the coronary arterial system. Coronary artery aneurysms and ectasia are significant complications of Kawasaki disease and develop in 15% to 25% of untreated children [131]. They may lead to ischemic heart disease or sudden death. The case fatality rate in KD in Japan is 0.08% [132]. The standardised mortality ratio in patients diagnosed between 1982 and 1992 was 1.25 (95% CI, 0.84 to 1.85) overall and 2.35 (95% CI, 0.96 to 5.19) for boys with cardiac sequelae [133]. 

In the United States, the in-hospital mortality rate is 0.17% (the investigators used administrative data that may include readmissions for coronary disease). Virtually all deaths in patients with Kawasaki disease result from its cardiac sequelae [134]. The peak mortality occurs 15 to 45 days after the onset of fever, during which time well-established coronary artery vasculitis occurs concomitantly with marked elevation of the platelet count and a hypercoagulable state [135]. However, sudden death from myocardial infarction (MI) can occur many years later in children and adults with coronary artery aneurysms and stenoses. Many fatal and non-fatal MI cases in young adults have now been attributed to “missed” KD in childhood [136]. Indeed, among adults under the age of 40 with suspected myocardial ischemia who underwent coronary angiography in San Diego, CA, 5% had lesions consistent with late sequelae of KD. 

### 7.3. Cardiac Histology

Amano et al. [137] studied the affection of arteries in KD, including coronaries, aorta, carotid, celiac, iliac, hepatic, splenic, mesenteric, renal, lumbar arteries, and the venous system. The most exciting discovery was that the coronary arteries are the vessels most affected, and that the development of the inflammatory process has six stages, as shown in Table 10, including degeneration of endothelial cells, edema and degeneration of the media, necrotizing pan arteritis, granuloma formation, scar formation, and aneurysm formation. It is characteristic that these six types of lesions are simultaneously observed not only in various areas of the arterial tree in the same patient but also in different portions of one artery. 

The wall of the aneurysm itself also shows various changes according to the duration of the disease. In the patients who died within one month, the vascular wall shows mostly necrotizing panarteritis. In longer-living patients, granulation tissue change replaces the acute inflammatory lesion. In cases of over two months duration, most of the vascular wall shows scar tissue change. Organisation or recanalization of the thrombus formed in the aneurysm is also seen [138].

### 7.4. Cardiac Immunohistochemistry and Immunofluorescence

At immunohistochemistry FC gamma receptor 1, 2 and 3, intravenous immunoglobulin (IVIG) and c-chemokine receptor type 2, in association with other factors listed in Table 1 are part of numerous detectable elements of Kawasaki Disease [139,140].

### 7.5. Genetic and Blood Markers

Genetic factors could play a decisive role in the pathogenesis of KD. Several additional findings support a genetic component to KD susceptibility, including the predilection for children of East Asian and Pacific Islander descent, even with transmigration [141]; ten times more risk of KD in siblings of patients with parental history of KD; and two-times higher risk in children with parental history of KD. Initial studies on the genetic basis of KD were focused on Human Leukocyte Antigen (HLA) and found HLA-DRB1, HLA B5, Bw51, and Bw44 to be associated with KD susceptibility. With the beginning of Genome-Wide Association studies, considerable progress has been achieved in identifying potential susceptibility loci. Several single-nucleotide polymorphisms (SNPs) in different genes and gene regions have been implicated in family linkage and genome association studies: caspase 3 (CASP3), inositol 1,4,5-trisphosphate kinase-C (ITPKC), CD40, FCGR2a, and B-cell lymphoid kinase (BLK) [128,142]. Interestingly, many of the SNPs associated with KD have been identified in other inflammatory diseases such as rheumatoid arthritis, ulcerative colitis, systemic lupus erythematosus, and systemic sclerosis. These findings indicate a common pathway in the inflammatory immune response.

## 8. Cardiopathy in Systemic Lupus Erythematosus (SLE)

Systemic lupus erythematosus (SLE) is a chronic autoimmune disease that affects multiple organs and tissues in the body. It is characterised by the production of autoantibodies against self-antigens, resulting in tissue damage and inflammation. SLE predominantly affects young women of childbearing age, with a female to male ratio of 9:1. The disease can present with a wide range of clinical features, including fever, fatigue, joint pain, skin rash, and kidney, heart, and lung involvement. SLE patients can also have non-specific symptoms such as weight loss, hair loss, and Raynaud’s phenomenon [143].

### 8.1. Pathogenesis

The exact cause of SLE is unknown, but it is believed to be triggered by a combination of genetic and environmental factors. Genetic studies have identified several susceptibility genes, including HLA-DR2, HLA-DR3, and PTPN22. Environmental triggers such as infections, drugs, and hormonal factors can also contribute to the development of SLE by promoting the activation of autoreactive T and B cells [143].

### 8.2. Cardiac Clinical Findings

Cardiac involvement is a common complication of SLE, with up to 50% of patients developing some form of cardiovascular disease. The most common cardiac manifestation is pericarditis, which presents with chest pain, fever, and ECG changes. Other cardiac manifestations include myocarditis, endocarditis, valve disease, and coronary artery disease [144].

### 8.3. Cardiac Histology

In patients with SLE, cardiac histology can reveal inflammation, fibrin deposition, and necrosis in the myocardium and pericardium. The histological features of SLE-associated myocarditis are like those of viral myocarditis, with mononuclear cells infiltrating in perivascular and interstitial tissues [145].

### 8.4. Cardiac Immunohistochemistry and Immunofluorescence

Immunohistochemical staining of endomyocardial biopsy specimens showed mild interstitial edema in almost the totality of cases, enriched by a wide presence of T lymphocytes and OKT 8 lymphocytes; besides at immunofluorescence IgG and fibrinogen deposition in the membrane of cardiac myocytes and in the interstitium can be detected [146]. 

Some samples of cardiac tissue show through immunohistochemical techniques an elevated concentration of MHR (Monocyte/High Density Lipoprotein Cholesterol Ratio), of high-density lipoproteins and E-selectin [134].

### 8.5. Genetic and Blood Markers

SLE has a strong genetic component, with heritability estimates ranging from 50% to 70%. Genome-wide association studies have identified more than 100 genes, including *IRF5*, *STAT4*, *CD24* and *BLK*. These genes are involved in inflammatory regulation and have been implicated in the pathogenesis of autoimmune dysregulation [147]. Patients with SLE can have a wide range of haematological abnormalities, including anaemia, leukopenia, and thrombocytopenia. The presence of antiphospholipid antibodies, anti-endothelial cell antibodies (AECA), anti-cardiolipin antibodies and antibodies against paraoxonase 1 can also lead to thrombosis and miscarriage in SLE patients. Autoantibodies against double-stranded DNA, Ro/La, and Sm can also be detected in the blood of SLE patients [148].

## 9. Cardiopathy in Rheumatoid Arthritis

Rheumatoid arthritis (RA) is a chronic autoimmune disease characterised by inflammation and destruction of synovial joints, leading to pain, stiffness, and functional disability. RA affects approximately 1% of the population worldwide, with women being more commonly affected than men. The disease can occur at any age but usually begins in middle age. The cause of RA is not fully understood, but it is believed to involve a combination of genetic and environmental factors [149,150].

### 9.1. Pathogenesis

The pathogenesis of RA involves the activation of immune cells, including T and B lymphocytes, macrophages, and dendritic cells, which infiltrate the synovial tissue and release cytokines, chemokines, and other inflammatory mediators. This inflammatory response leads to synovial hyperplasia, angiogenesis, and pannus formation, which can invade and destroy cartilage and bone. RA is also associated with the production of autoantibodies, including rheumatoid factor (RF) and anti-citrullinated protein antibodies (ACPAs), which are thought to contribute to the disease process [151].

### 9.2. Cardiac Clinical Findings

Patients with RA are at increased risk for cardiovascular disease (CVD), including myocardial infarction, heart failure with left ventricle dilatation, coronary arteritis and stroke. The risk of CVD in RA is believed to be due to a combination of traditional cardiovascular risk factors, such as hypertension, hyperlipidemia, and smoking, as well as RA-specific factors, such as inflammation and disease activity. RA patients may also develop pericarditis, myocarditis, and valvular disease [152].

### 9.3. Cardiac Histology

Histological examination of cardiac tissue from RA patients with CVD reveals evidence of inflammation, including infiltration of immune cells, cytokine expression, and oxidative stress. Inflammatory infiltrates, such as macrophages, monocytes, T-cells and B-cells, can be found in the myocardium, pericardium, and valves. These changes can lead to myocardial apoptosis, fibrosis, and dysfunction, as well as valvular thickening and insufficiency [153].

### 9.4. Cardiac Immunohistochemistry and Immunofluorescence

Immunohistochemical analysis of cardiac tissue samples show the presence of TNFRI factor, while also PAD1 in cytoplasmic granules, PAD 2 in leukocytes, PAD 3 and PAD 4 in cardiomyocytes can be detected. PAD genes stand for peptidyl arginine deiminase enzymes [154].

### 9.5. Genetic and Blood Markers

RA has a strong genetic component, with genetic factors accounting for up to 50% of disease susceptibility. The major genetic risk factor for RA is the human leukocyte antigen (HLA) class II locus, particularly the *HLA-DRB1* gene. The presence of specific *HLA-DRB1* alleles, such as *HLA-DRB101* and *HLA-DRB104*, is associated with an increased risk of developing RA. Other genetic factors, including polymorphisms in cytokine genes and genes involved in immune regulation, such as *PTPN22*, *DRB1* and *ACPA*, have also been implicated in the development of RA [155]. Blood tests used in the diagnosis and monitoring of RA include the rheumatoid factor (RF) and anti-citrullinated protein antibody (ACPA) tests. RF is an antibody that targets the Fc portion of IgG and is found in approximately 70% of RA patients. ACPAs are a more specific marker of RA and are present in approximately 60% of patients. Increased levels of TNF-a, TNFRII messenger RNA, CD4 and CD 28 lymphocytes, IL-12 and anti-CD28 antibodies can also be detected in the blood of RA patients.

## 10. Cardiopathy in Systemic Sclerosis

Systemic sclerosis, also known as scleroderma, is a chronic autoimmune disorder that affects the connective tissue in various parts of the body, including the skin, blood vessels, and internal organs. The hallmark of systemic sclerosis is excessive collagen deposition, leading to fibrosis and thickening of affected tissues. This can result in a range of clinical manifestations, including skin changes, joint pain, digestive problems, and cardio-pulmonary hypertension [156].

### 10.1. Pathogenesis

The exact cause of systemic sclerosis is unknown, but it is thought to involve a complex interplay between genetic and environmental factors. Dysregulation of the immune system and abnormal activation of fibroblasts are believed to contribute to the development of the disease. These processes can lead to increased production of extracellular matrix proteins, such as collagen, and subsequent fibrosis [157].

### 10.2. Cardiac Clinical Findings

Cardiac involvement is common in systemic sclerosis and can manifest in various ways, including myocardial fibrosis, arrhythmias, pericarditis, and congestive heart failure. Pulmonary arterial hypertension is also a significant complication of systemic sclerosis, which can lead to right heart failure and increased mortality [158].

### 10.3. Cardiac Histology

Myocardial fibrosis is a hallmark of cardiac involvement in systemic sclerosis, with activated T-Lymphocytes and macrophage infiltration. This increased collagen deposition leads to thickening and stiffening of the heart muscle. This can result in impaired diastolic function, leading to heart failure with preserved ejection fraction. In more severe cases, fibrosis can also involve the conduction system, leading to arrhythmias and heart block [159].

### 10.4. Cardiac Immunohistochemistry and Immunofluorescence 

With the support of an avidin-biotin-immunoperoxidase method, immunohistochemical analysis can detect a positivity for CD3, CD68, HLAII factor and SM-actin [160].

### 10.5. Genetic and Blood Markers

Genetic factors are thought to contribute to susceptibility to systemic sclerosis, with several genes implicated in disease pathogenesis. These include genes involved in immune regulation, as well as genes involved in fibrosis and angiogenesis, such as *IRF4*, *IRF55*, *TNFAIP3*, *TNFSF4*, *PTPN22*, *BANK1*, and *IL-21* gene [161]. Various biomarkers have been identified in systemic sclerosis, including autoantibodies such as anti-nuclear antibodies and anti-centromere antibodies, which are associated with distinct clinical subsets of the disease. Elevated levels of cytokines such as IL-6, IL-12, IL-23, and tumour necrosis factor-alpha have also been found in the blood of affected individuals, suggesting a role for immune dysregulation in disease pathogenesis.

## 11. Cardiopathy in Sjogren Syndrome

Sjogren syndrome is a chronic autoimmune disorder characterised by lymphocytic infiltration of exocrine glands, particularly the salivary and lacrimal glands, resulting in dry mouth and eyes. In addition to glandular dysfunction, Sjogren Syndrome can also cause systemic symptoms such as fatigue, joint pain, and skin rashes. Sjogren Syndrome can occur alone, or in combination with other autoimmune disorders such as rheumatoid arthritis or lupus [162].

### 11.1. Pathogenesis

The exact cause of Sjogren syndrome is unknown, but it is believed to involve a combination of genetic and environmental factors. Immune dysregulation is thought to play a key role in the development of the disease, with infiltration of lymphocytes into exocrine glands resulting in tissue damage and dysfunction. In addition, autoantibodies against various cellular components are commonly found in affected individuals, further contributing to the immune-mediated damage [163].

### 11.2. Cardiac Clinical Findings

Cardiac involvement is relatively uncommon in Sjogren syndrome but can occur in some cases. The most common cardiac manifestation is conduction abnormalities, such as atrioventricular block or bundle branch block. Pericarditis, vasculitis, and valvular insufficiency may occur. In more severe cases, cardiomyopathy, and heart failure with diastolic left ventricular dysfunction can be a cardiac representation of Sjogren Syndrome [164].

### 11.3. Cardiac Histology

Cardiac histology in Sjogren syndrome is not well characterised, but some studies have suggested that myocardial inflammation and fibrosis may be present in affected individuals with cardiac involvement, with leukocytoclastic and macrophage infiltration.

### 11.4. Cardiac Immunohistochemistry and Immunofluorescence 

Immunohistochemical studies have identified lymphocytic infiltration in affected exocrine glands, with predominance of CD4+ and CD45+ T cells and B cells. Autoantibodies against various cellular components, such as Ro and La antigens, are commonly found in affected individuals and are thought to contribute to immune-mediated damage, as well Siglec-1 found in macrophage in cardiac septal region [165].

### 11.5. Genetic and Blood Markers

Sjogren syndrome has a strong genetic component, with several genes implicated in disease susceptibility. These include HLA genes (*HLA-DR3*), as well as genes involved in immune regulation and cytokine signalling. *Id3* pathway may be involved, because some authors have found that *Id3* knockout mice develop many symptoms like those found in Sjogren’s syndrome. Various autoantibodies are commonly found in the blood of individuals with Sjogren syndrome, including anti-Ro and anti-La antibodies. In addition, elevated levels of inflammatory cytokines such as IL-2, IL-6, IL-1, IFN-gamma, and tumour necrosis factor-alpha have been reported in affected individuals. Some patients can present C3 or C4 hypocomplementemia [166].

## 12. Cardiopathy in Polymyositis and Dermatomyositis

Polymyositis and dermatomyositis are chronic autoimmune disorders characterised by inflammation and weakness of the muscles, particularly the proximal muscles of the arms and legs. Dermatomyositis is also associated with skin rashes and other skin changes. These disorders can occur at any age but are most seen in middle-aged and older adults [167].

### 12.1. Pathogenesis

The exact cause of polymyositis and dermatomyositis is unknown, but they are believed to involve a combination of genetic and environmental factors. Immune dysregulation is thought to play a key role in the development of the disease, with infiltration of lymphocytes and other immune cells into affected muscles resulting in tissue damage and dysfunction. In addition, autoantibodies against various cellular components are commonly found in affected individuals, further contributing to the immune-mediated damage [168].

### 12.2. Cardiac Clinical Findings

Cardiac involvement can occur in polymyositis and dermatomyositis, although it is relatively uncommon. Cardiac manifestation can include coronary arteritis, valvular insufficiency or stenosis, and conduction abnormalities. In more severe cases, cardiomyopathy, cardiac ischemia, and heart failure can occur [155].

### 12.3. Cardiac Histology

Cardiac histology in polymyositis and dermatomyositis is not well characterised, but some studies have suggested that active myocarditis with lymphocytic infiltration, focal fibrosis, vasculitis, intimal proliferation, medial sclerosis of vessels and fibrosis of the sinoatrial node may be present in affected individuals with cardiac involvement.

### 12.4. Cardiac Immunohistochemistry and Immunofluorescence 

Immunohistochemical studies have identified lymphocytic infiltration in affected muscles, with predominance of CD8+ T cells. Autoantibodies against various cellular components, such as anti-Jo-1 antibodies, are commonly found in affected individuals and are thought to contribute to immune-mediated damage [169].

### 12.5. Genetic and Blood Markers

Polymyositis and dermatomyositis have a strong genetic component, with several genes implicated in disease susceptibility. These include HLA genes, as well as genes involved in immune regulation and cytokine signalling.

Various autoantibodies are commonly found in the blood of individuals with polymyositis and dermatomyositis, including anti-Rho-1 and anti-SAE antibodies in polymyositis and anti-Mi-2, anti-MDA5, anti-NXP2, and anti-TIF1 antibodies in dermatomyositis. In addition, elevated levels of inflammatory cytokines such as interleukin-6 and tumour necrosis factor-alpha have been reported in affected individuals. Elevated serum levels of creatine kinase MB isoenzyme may be found in patients with polymyositis [170].

## 13. Cardiopathy in Acute Rheumatic Fever 

Acute rheumatic fever (ARF) is an autoimmune disease triggered in some children and young adults by infection with group A streptococci. The incidence is 8 to 51 per 100,000 people worldwide. Overcrowding and poor socioeconomic conditions are directly proportional to the incidence of ARF.

The diagnostic criteria for ARF have been constantly updated to improve the sensitivity. The diagnosis was entirely clinical however, recently echocardiographic evidence has been added as a major criterion. The following table (Table 11) shows the 2015 updates of Jones criteria which also summarise the main clinical features [171].

### 13.1. Pathogenesis

ARF occurs due to an autoimmune response initiated by GAS pharyngitis. The Streptococcal antigens, such as M protein and carbohydrate antigen (N-acetyl-beta-D glucosamine), produce antibodies which cross react with human cardiac proteins such as myosin and laminin, and result in humoral mediated injury [171].

### 13.2. Cardiac Clinical Findings

ARF and its sequelae rheumatic heart disease (RHD) remain significant causes of cardiovascular morbidity and mortality [172]. Cardiac involvement occurs in the form of carditis, which can be variable in severity. ARF may resolve entirely or persist and evolve into a chronic rheumatic heart disease (RHD) process.

Carditis is an early manifestation, with 80% of patients developing it in the first two weeks of ARF. Tachycardia is an early sign of carditis. The mitral valve is the commonest involved, and mitral regurgitation (MR) is the most joint abnormality. Aortic regurgitation can also occur but usually occurs in association with MR. The severity of carditis may be variable, ranging from an asymptomatic patient (mild MR) to a critically ill patient with dyspnea, palpitations, and heart failure (ruptured chordae causing acute severe MR) [173,174]. Although classically described as “pancarditis”, endocarditis is the most dominant involvement. Pericarditis occurs in 4% to 11% of ARF patients and usually resolves without sequelae.

The valve damage during an ARF episode may persist or is accentuated by recurrent episodes of ARF. MR results in compensatory dilation of the left atrium (LA) and left ventricle (LV) to maintain cardiac output, resulting in a prolonged asymptomatic period. However, the MR progresses over time due to progressive LV enlargement, which explains the phrase “MR begets MR”. Eventually, LV dysfunction sets in when the patient becomes symptomatic. As the disease progresses, LA dilatation and pulmonary venous Hypertension develop, resulting in exertional dyspnea, paroxysmal nocturnal dyspnea, and orthopnea, with severe LA outflow obstruction. With severe LA outflow obstruction, pulmonary artery hypertension results, and signs of right heart failure develop [175].

### 13.3. Cardiac Histology

In the acute phase of ARF, focal inflammatory lesions can be found in various tissues. Characteristic lesions develop in the heart, called Aschoff bodies, which comprise foci of T lymphocytes, some plasma cells, and large macrophages with an activated appearance called Anitschkow cells (pathognomonic for RF). These macrophages have an abundant cytoplasm and a round or ovoid (sometimes binucleated) nucleus, in which the chromatin condenses into a thin, wavy central ribbon (hence the name “caterpillar cells”).

Inflammation of the endocardium and left valves typically results in fibrinoid necrosis within the tendinous cusps or chordae [175].

### 13.4. Cardiac Immunohistochemistry and Immunofluorescence

Immunohistochemistry staining techniques highlight T cell recruitment, leading to granulomatous inflammation and Aschoff body formation, which is the pathologic hallmark of RHD. CD4+ T cells are the primary effectors of this process, leading to chronic RHD development [176].

### 13.5. Genetic and Blood Markers

Genetic factors such as HLA class II alleles (DR, DQ), specific B cell alloantigen (D8/17), and genetic polymorphisms (TNF-alfa, interleukins, TGF B1) have been proposed to contribute to this host susceptibility.

The most common autoantibody type found in individuals with ARF’s blood is anti-Streptococcus A [176].

## 14. Cardiopathy in Sarcoidosis

Sarcoidosis is a systemic inflammatory disorder characterised by the presence of non-caseating granulomas in affected organs, most commonly the lungs, lymph nodes, and skin. The disease can affect individuals of any age, race, or sex, and has a variable clinical course ranging from self-limited to chronic and progressive [177].

### 14.1. Pathogenesis

The exact cause of sarcoidosis is unknown, but it is believed to involve a combination of genetic and environmental factors. Immune dysregulation is thought to play a key role in the development of the disease, with activation of T lymphocytes and macrophages leading to granuloma formation and subsequent tissue damage. Genetic factors, including polymorphisms in genes involved in immune regulation and antigen presentation, are also thought to contribute to disease susceptibility [178].

### 14.2. Cardiac Clinical Findings

Cardiac involvement can occur in sarcoidosis, although it is relatively uncommon. The most common cardiac manifestation is conduction abnormalities, such as atrioventricular block or bundle branch block. In more severe cases, cardiomyopathy and heart failure can occur [179].

### 14.3. Cardiac Histology

Cardiac histology in sarcoidosis is characterised by the presence of non-caseating granulomas in affected tissues, which can lead to inflammation, fibrosis, and scarring. These granulomas are composed of T lymphocytes and macrophages and can be identified on biopsy specimens [180].

### 14.4. Cardiac Immunohistochemistry and Immunofluorescence

Immunohistochemical samples of the heart in patients with sarcoidosis where heart was involved show a mild increase of CD4, CD8, CD15, CD20, CD68 factors, associated with a frequent negativity for IL6-, S100- and Ki67- [181,182].

### 14.5. Genetic and Blood Markers

Sarcoidosis has a vital genetic component, with several genes implicated in disease susceptibility. These include genes involved in immune regulation and antigen presentation, such as HLA genes (HLA-DRB*0301) and genes encoding components of the major histocompatibility complex [183].

Various blood tests can be used to evaluate individuals with sarcoidosis, including measuring serum angiotensin-converting enzyme levels, which are often elevated in affected individuals. In addition, inflammatory markers such as C-reactive protein and erythrocyte sedimentation rate may be elevated in active disease. Various no-specific markers are commonly found in the blood of individuals with sarcoidosis, including Rheumatoid factor (RF) and Antinuclear antibodies (ANA) [184].

## 15. Mis-C COVID Related Cardiopathy

MIS-C stands for Multisystem inflammatory syndrome in children; it is considered a considerably worrying health condition that could be directly linked to SARS-CoV-2 infection [185]. This disease was first described as early as the spring of 2020, during the first wave of the COVID-19 pandemic, when it was misdiagnosed or confused with Kawasaki Disease (KD). This pathology shares a wide range of clinical features with MIS-C [186]. 

MIS-C develops at later stages during Severe Acute Respiratory Syndrome (SARS-CoV-2); thus, after the appearance of typical COVID-19 symptoms such as fever, dry cough, sore throat, cutaneous rash, conjunctivitis, and anosmia, some children started developing a severe multiorgan dysfunction, including cardiac, renal, respiratory, hematologic, gastrointestinal, and neurological symptoms; more than 20% of these patients even suffered acute hypotension or shock which required immediate action in order to avoid the further worsening of their health [187].

Clinical guidelines for these conditions refer to a patient’s age below 21. The median age for children who suffer from this condition is between 8–11 years old (range 1–20 years); other authors describe cases of MIS-C in adults ages 20 to 30, who met all the criteria defining the disease except for age itself. 

As far as cardiac involvement, it was described as having a role in the rapidly declining health condition of some children, where acute myocardial dysfunction or systemic hyperinflammation-vasodilation, could be traced as the main factors contributing to the development of hypotension or shock, which required intensive care treatment in 6–10% of young patients. Other cardiac symptoms include coronary artery aneurysms in 6–24% and arrhythmia in 7–60% of children with MIS-C.

MIS-C diagnosis is based on clinical evidence with a recent history of SARS-CoV-2 infection, Kawasaki-like symptoms, age-related (1–20 years old), and laboratory findings [188,189,190,191].

### 15.1. Pathogenesis

MIS-C is strongly linked to SARS-CoV-2 infection, and it is a sequel of the disease in a paediatric population. Pulmonary manifestation of SARS-CoV-2 is less frequent in children than adults; this is probably due to the lower expression of the ACE-2 receptor (angiotensin-converting enzyme receptor) at a young age; ACE-2 receptor is renowned for being a main target for SARS-CoV-2 entrance into pulmonary cells [192,193,194,195,196,197,198]. This has led some authors to speculate that MIS-C may be characterised by a delayed immunological and inflammatory response after symptomatic or asymptomatic COVID-19 infection [185,199].

### 15.2. Cardiac Clinical Findings

The main cardiac structure involved and clinical picture correlated are summarised in Table 12 [200,201].

### 15.3. Cardiac Histology

There is no autopsy data referring to MIS-C patients, and no hematoxylin-eosin staining pools analyses could be found in the literature.

In vivo data suggest that considering only heart involvement, we can divide MIS-C patients into four subgroups: patients with only coronary artery lesions, patients with solely myocardial involvement, patients with involvement of both, and patients without manifest cardiac involvement [202]. 

Patients with coronary artery lesions had Kawasaki-like features such as younger age, thrombocytosis, and normal ferritin levels, without giant coronary artery aneurysms and signs of myocardial infarction nor shock; thus, it is possible to trace back a common histologic pattern between Kawasaki Disease and this peculiar presentation of MIS-C.

Patients with both previously mentioned are very similar to the patients with only myocardial infiltration for what concerns clinical presentation but are also characterised by less extensive thrombocytopenia. Patients without cardiac involvement have a rather high rate of shock and ICU admission. 

### 15.4. Cardiac Immunohistochemistry and Immunofluorescence 

While no data about cardiac immunohistochemistry is widely available, blood markers have been studied for the disease. We can detect a general increase in inflammatory blood markers, such as CRP (C Reactive Protein) and ESR (Erythrocyte Sedimentation Rate), in all groups of patients.

In the group of patients with just coronary artery lesions, a form of leukocytosis and thrombocytosis very similar to the one detectable in KD can be seen. 

### 15.5. Genetic and Blood Markers

Dolhikoff et al. [203], in their case report about a child with post-COVID-19 MIS-C, refer to a diffuse myocardial interstitial inflammation at cardiac histology with hematoxylin-eosin staining containing lymphocytes, macrophages, a few neutrophils, eosinophils, and foci of cardiomyocyte necrosis. Foci of myocardial necrosis indicated by C4d staining were detected, while in other myocardial interstitial inflammation fields, other foci containing CD68+ and CD45+ cells were found.

Data are in continuing update about the immunohistochemistry of MIS-C patients; neutralising autoantibodies against Type I IFNs are frequently observable and are often associated with a worse outcome of the disease, while other antibodies against self-antigens have been described, often present with a slightly different pattern in every patient [204].

An increased expression of type I and type II interferons, *STAT1*, *IRF3*, *IRF7* monocyte gene, NF-κB, and NK natural killer has been detected [205].

In patients with isolated myocardial injury, thrombocytopenia, hypoalbuminemia, and hypoproteinemia are predominant and indicate a severe hepatic dysfunction, such as the highest level of troponin, ferritin, ALT/AST, LDH, and D-dimer.

In the group of patients without any cardiac involvement, serum ferritin, CRP, and D-dimer are moderately increased.

## 16. Coxsackieviruses B1-B5 Cardiopathy

Coxsackieviruses are a group of RNA viruses belonging to the family of Enteroviridae that can be divided into two subgroups—coxsackievirus A and B—and comprise more than 50 serotypes, each of which can cause a different clinical syndrome.

The most common clinical syndromes associated with coxsackie infections are summer grippe, hand-foot-mouth disease, and herpangina, but also severe diseases such as aseptic meningitis and myocarditis [206].

Coxsackieviruses serotype from 1 to 5, especially the B3 form, are implicated in the latter; heart failure in the neonatal period secondary to uterine myocarditis and over 20% of adult cases of myocarditis and dilated cardiomyopathy are associated with Group B infections [207]. In the U.S., approximately five million enteroviral infections are attributed to CVB1-5, and about 12% of them may have myocardial involvement in which CVB1, CVB3, and CVB5 serotypes are usually involved [208].

### 16.1. Pathogenesis

Each serotype belonging to the coxsackievirus strain has its pathogenetic aspects; first, we must consider specific interactions between the virus and target receptors. 

Among the factors involved in pathogenesis, we recognize two proteinic receptors as Coxsackievirus-adenovirus receptor (CAR) and the Decay accelerating factor (DAF) [209].

CAR is expressed in intercalated discs that link and mediate signals between myocardial cells, while DAF is a standard marker expressed in endothelial and epithelial cells. 

The expression of DAF in other tissue, like the Central nervous system (CNS), can help neuronal infections by hematogenous spread or axonal transport; such neurons undergo a process of viral replication and activation of an autoimmune response that can cause extensive neuronal damage [210].

### 16.2. Cardiac Clinical Findings

Coxsackievirus B1-5 has a specific tropism for the myocardium. Viral molecules have a lytic cycle infection inside myocardial cells. Thus, after entering host cells, virions multiply and release a progeny that exits infected hosts to infect other myocardial cells: this explains the inflammatory response of the organism against tissue threat posed by the virus, the development of myocarditis, and consequent cardiac damage and modulation [211]. Another important protein is 2ª protein that cleaves cytoskeletal dystrophin and dystrophin-associated glycoproteins [212]; dystrophin is a molecule frequently critical for connecting with the contractile protein F-actin inside cardiomyocytes. Besides, dystrophin deficiency because patients who develop dilated cardiomyopathy after infection with Coxsackie B viruses show a lack of dystrophin production [213].

### 16.3. Cardiac Histology

Myocarditis linked to coxsackie B infection comprises a lymphocytic mononuclear inflammatory infiltrate with cardiomyocytic damaged elements; these elements are part of a Th-2, Th-17 lineage [214].

### 16.4. Cardiac Immunohistochemistry and Immunofluorescence

Data coming from heart biopsies shows the positivity for VP-1 protein of the viral capsid, detected by means of RT-PCR, and the same molecule can be identified at immunofluorescence essays; genetic analyses detected that CVB-1 and CVB-3 sequences mutations of VP-1 protein are the most prominent [215].

### 16.5. Genetic Findings and Blood Markers

No specific findings about correlations between coxsackievirus infections and heart disease have been demonstrated as of now. Speculations about the role of some sorts of genetic determinants and coxsackieviruses (especially B strains) induced myopathies have been made [216] while it is certain the role of genes associated to type 1 diabetes mellitus and susceptibility to coxsackieviruses infections [217].

## 17. Epstein-Barr Virus Cardiopathy

Epstein-Barr virus (EBV) is a DNA virus belonging to the family of Herpesviridae, which usually infects more than 90% of the general population before adult age [218,219].

The type of pathology that could derive from EBV infection is very variable and depends on factors such age the age of the patient and his immunity condition; typically, in children, we record especially asymptomatic forms or mild pharyngitis, while in adolescents and adults, we witness a mononucleosis-like syndrome, or, in case of immunocompromised patients, a lymphoproliferative disease.

The incubation period after infection is 4–6 weeks, which could lead to asymptomatic, mild symptomatic disease of infectious mononucleosis.

In the case of symptomatic forms, they usually last 2–4 weeks, but in some patients, asthenia could last for more than six months, while 2–3 weeks after infection, lymphocytosis is present with more than 10% of atypical lymphocytes. In the case of the development of infectious mononucleosis, which occurs in around 20–25% of adolescents and young adults with EBV, the disease is preceded by prodromal like asthenia, myalgias, and physical unrest that can last 1–2 weeks before the appearance of fever, lymphadenitis, pharyngitis, and 2–3 weeks after infection, splenomegaly [220].

### 17.1. Pathogenesis

EBV infects the oropharyngeal and salivary gland epithelium’, as long as B-cell lymphocytes of tonsillar crypts, before a subsequent viremic period. It is generally transmitted by contact with oral secretions through mediums like saliva, while oropharyngeal secretions eliminate it.

The virus generates a polyclonal activation of B-cells, while, during acute infective processes, lymphocytes T reactive cells proliferate. Besides, B memory cells represent the essential infective tank [221].

Cell-mediated immunity is more critical than humoral immunity for infection control; if immunity perpetrated by T lymphocytes is compromised, infected B-cells could proliferate, progressing toward a neoplastic transformation [222].

### 17.2. Cardiac Clinical Findings

Cardiac involvement during EBV infection is relatively rare. Even so, reports about cardiac complications after EBV infections are present; as part of herpesviruses, EBV usually causes recurring and persistent infections that could lead to myocarditis. Myocarditis is a rare complication of acute or chronic EBV infection. It is usually caused by a direct viral infection and toxic and autoimmune mechanisms, where autoantibodies activate the complement or cause cellular cytotoxicity [223]. In these cases, an acute myocardial involvement is followed by a latent phase that could lead to necrosis of myocardial cells due to an autoimmune reaction against virus-altered myocytes. In contrast to this process, another part of the myocardium dilates in compensation, causing functional problems such as mitral or tricuspid insufficiency and atrial dilation. Eventually, the dilation of cardiac chambers results in dilated cardiomyopathy. It can assist the development of thrombi, while in parallel with left atrium dilation, arrhythmias such as atrial fibrillation usually occur [224]. Furthermore, sudden death can occur at any stage during the development of dilated cardiomyopathy [225].

Patients affected by cardiac involvement during EBV infection refer to symptoms such as heart failure, progressive exertional dyspnea with progressively lower exercise tolerance, paroxysmal nocturnal dyspnea, and peripheral edema [226]. 

Chest pain may be present in the case of pericardium involvement, which is rare in immunocompetent patients [227].

### 17.3. Cardiac Histology

Myocardial infection by Epstein–Barr virus (EBV) has been associated with inflammatory cardiomyopathy because of its toxic effect on cardiomyocytes. It is linked to cardiac syndrome X because of its tropism for the endothelial cells of small coronary vessels [228,229]. 

The comprehensive inflammatory response in the case of myocardial infection often gives the reason for the development of focal myocarditis with rare necrotizing vasculitis of the intramural vessels and the presence of a CD4+ and CD8+ lymphocytic infiltrate, besides the occurrence of vasculitis in intramural vessels can contribute to localised ischemia of the ventricular wall, with subsequent infarction and eventual dilation of the ventricular chamber, with the abundant intervention of monocytes, macrophages, and fibroblasts infiltrate.

### 17.4. Cardiac Immunohistochemistry and Immunofluorescence 

The only immunohistochemical factor which is described in the literature and is specific to cardiac myocardium involvement is EBV latent membrane protein 1, a strong positivity in both cardiomyocytes and inflamed vessels; the same goes for IL-1, IL-6, and IL-17, which are above-range.

### 17.5. Genetic and Blood Markers

It has been cleared that different genetic etiologies converge to the EBV induced disease. Various genes have been studied in regard of a possible link with susceptibility to infection by EBV, but none of them has revealed a specific correlation with cardiac tropism of the virus and cardiocirculatory symptoms [230].

- *MAGT1*, *ITK*, *RASGRP1*, *CTPS1*, *CD27*/*CD70*, *TNSRSF9*, *CARMIL2*, are associated with anomalous proliferation of CD8+ cells.
- *SH2D1A*, *MAGT1*, *CD27*/*CD70*, *TNSRSF9*, *RASGRP1*, *CARMIL2*, are associated with anomalous activation of cytotoxic pathways in CD8+ T cells, NK cells in response to Ab-presenting B cells.
- *DOCK8*, *STK4*, *CORO1A*, *CARMIL2*, *PIK3CD*-*GOF*, defect in cytoskeletal rearrangement in EBV infected cells, CD8+ T cells and NK cells.
- *DOCK8*, *STK4*, *CORO1A*, *PIK3CD*-*GOF*, are linked with loss of naïve CD8+ T cells.

Considering the concomitant EBV infection in these patients, increased VCA IgM, VCA IgG, and EBV-EA IgG values can be detected in blood samples. Among other blood markers persistent are the detection of leukopenia, while inflammatory blood markers such as CRP are usually elevated; furthermore, a specific marker of heart involvement during EBV infection is Troponin I, elevated in case of myocardial necrosis.

## 18. Herpes Simplex Cardiopathy

The Herpes simplex virus (HSV) is a group of viruses belonging to Herpesviridae family, which also comprehend Varicella zoster virus (VZV), Epstein-Barr virus (EBV), Cytomegalovirus (CMV), Human herpesvirus-6 (HHV-6), Human herpesvirus 7 (HHV7) and Human herpesvirus 8 (HHV8).

The classical HSV serotypes are HSV-1 and HSV-2, which differentiate for presenting different patterns of antigenic expression, and different features of growth in culture and clinical aspects; HSV-1 is the infective agent in about 80–90% of labial or oral mucosal infections [231]. Conversely, HSV-2 is responsible for 70–90% of urogenital infections [232]. The transmission is by direct contact with oral o genital secretions.

In almost all patients, the virus enters a period of latency after a primary infection. At the same time, in 60–90% of them, we witness a viral reactivation after unchaining events such as emotional solid stressing events, the prolonged exposure to sun rays, and traumas.

HSV has rarely been found in specimens of endomyocardial biopsies of patients (approximately less than 1%) with acute pericarditis and myocarditis [233,234].

### 18.1. Pathogenesis

The virus initially infects parabasal and intermediate cells of the epithelium; following viral replication, we assist in the degeneration of infected cells with the formation of multinucleate elements and lithic processes in cells. The infection is propagated by direct contact that leads to the formation of the herpetic vesicle on an inflammatory basis; primary infection often induces a specific inflammatory response, with an intralesional accumulation of activated and cytotoxic lymphocytes, macrophages, and local cytokines and chemokines production [235].

After primary infection begins the latency period, characterised by the localization of the virus in sensitive ganglia afferent to the cutaneous lesion, probably after centripetal diffusion along the sensitive nerve [236].

### 18.2. Cardiac Clinical Findings

HSV has rarely been reported to cause pericarditis or myocarditis in immunocompetent patients. In most cases where the patients presented with a cardiovascular disease caused by HSV infection, whose viral proteins were found in myocardial tissue, there was often a correlation between a condition of immunosuppression at the basis of the pathology [237].

Myocardial infection in these patients could lead to the development of a severe form of acute or even fulminant myocarditis, indistinguishable from prodromes and symptoms from other viral and non-viral myocarditis, capable of causing rapid death of the patient [238]. 

### 18.3. Cardiac Histology

Results from simples, coloured by hematoxylin-eosin, coming from endomyocardial biopsies or autopsies show processes of necrotizing inflammation, inflammatory cells are frequently highlighted around fibres of the conduction system, thus providing additional material of study for possible implications of HSV infections with arrhythmias. 

### 18.4. Cardiac Immunohistochemistry and Immunofluorescence 

Immunohistochemistry allows us to define the type of infiltrate resulting in myocardial tissues during an active HSV infection; in this direction, those techniques detect anti-CD4 lymphocytes at interstitial and perivascular levels, while immunofluorescence against CD45 detects the presence of many leukocytes around small myocardial vessels and fibres of the conduction system [239].

### 18.5. Genetic and Blood Markers

No specific gene has shown a predictable correlation between HSV infection and cardiovascular complications. Genetic host variability has been described as one of the main factors leading to a variable response in patients after HSV-1 or two infections, especially for concerns gene families belonging to chromosomes 1, 6, 12, or 19, which encode for regulators of innate and adaptive immunity; among them, Moraru et al. [240] recognize MHC class I allotypes B*18, C*15, the group of alleles encoding A19 the high-affinity receptor/ligand pair *KIR2DL2/HLA-C1*, and the *CD16A-158V/F* dimorphism give an essential role in the host response against herpetic infection thanks in adaptive immunity and surveillance of its subversion. They also confirm the crucial role of cytotoxic cells (CTL and NK) and the contribution of genetic diversity to the clinical course of HSV-1 infection. No author has published material about blood markers in the case of HSV-related myocarditis, except for an increase in the common inflammatory markers such as CRP and ESV7.

## 19. COVID-19 Induced Cardiopathy

COVID-19 disease, also known as SARS-CoV-2, severe acute respiratory syndrome coronavirus 2, is an infectious viral pathology caused by a virus member of the coronaviridae family.

Symptoms of COVID-19 are variable and include headache, cough, fever, fatigue, shortness of breath, loss of smell, and test, that in about 15% further develop into severe symptoms such as dyspnea, hypoxia, and interstitial pneumonia, and in 5% of cases acute symptoms like respiratory failure, shock and multiorgan dysfunction which eventually lead to death in approximately 1% of cases; older people with previous pathologies are at higher risk for fatal consequences of COVID-19 [241].

### 19.1. Pathogenesis

The COVID-19 virus is usually transmitted via the respiratory airways due to the inhalation of droplets and airborne particles of aerosol after breathing, talking, coughing, and sneezing; infection can be facilitated in indoor spaces, by close contact with infected individuals and in particular conditions of temperature and humidity. After inhalation, the virus binds with its spike protein to specific molecules on the surface of host cells thanks to its high affinity to the receptor of angiotensin-converting enzyme 2 (ACE2) [242].

### 19.2. Cardiac Clinical Findings

Among the many clinical manifestations involved in covid infections, cardiac symptoms are frequent, especially in patients admitted to ICU. Elevation of troponin, hypoxemia, and left ventricular function depression are often present. On the other hand, modifications of endothelial hemostasis can contribute to arterial and venous thrombosis, resulting in potentially fatal damages, thus justifying the use of anti-thrombotic and anti-platelet drugs in the acute setting [243].

Other patients suffered myocardial inflammation and infection concomitant with the development of SARS-CoV-2 pathology or in the aftermath, especially in those who experienced the condition known as “long covid” [244].

Another challenge is represented by arrhythmias, both new or already diagnosed; those arrhythmias are often mediated by the systemic inflammatory response and other acute events such as ischemia, thrombosis, hypoxemia, and embolism [245].

### 19.3. Cardiac Histology

Though the death of myocarditis during SARS-CoV-2 infection is relatively uncommon, the cases of virus-positive patients who underwent autopsy after death describe a wide area of myocyte necrosis, with the presence of lymphocytic infiltration of the pericardium and perivascular area [246]. The study of those infiltrates shows a significant presence of CD3, CD8 and CD68 lymphocytes. Besides, some authors highlight thrombotic processes inside heart tissues [247].

### 19.4. Cardiac Immunohistochemistry and Immunofluorescence

Immunohistochemical stains usually detect an increase in CD3, CD8, and CD68 factors; the relation CD4/CD8 is usually <1, while only in a few cases do authors describe a positivity for CD410. Immunohistochemistry for ACE-2 detects the presence of this binding molecule with an interstitial and perivascular pattern, while TNFα, caspase 3, and IL-6 molecules, frequently overexpressed in fatal cases of SARS-CoV-2 get detected in the perivascular zone of the myocardial interstitial. Intercalated disc proteins such as N-cadherin, connexin 43 (Cx43), and NaV1.5m are localised lateralized inside myocyte cytoplasm during myocarditis processes [248].

### 19.5. Genetic Findings and Blood Markers

It demonstrates a specific correlation between genetic susceptibility and COVID-19; as of now, no specific genetic link between the infection and heart complications has been found. Interferons, for their role in the signalling cascade, play an essential part in the autocrine and paracrine mechanisms that lead to organism response against the virus. Among those factors, IFN-stimulated genes such as *ISGs*, *OAS1*, *OAS2*, and OAS3 give a general susceptibility to the development of coronavirus-related disease [249]. Other genetic factors implicated are *TLR3*, *TLR7*, and genes that encode for cytoplasmic cytokines and chemokines like CCL2, CCL3, CCL7 and CXCL10

## 20. Varicella Zoster Virus Cardiopathy

Varicella zoster virus (VZV) is a double strand DNA virus belonging to the Herpesviridae family, with a pathogenic cycle like the one of HSV. Disease manifestations of VZV, also known as Human herpes virus 3 (HHV3) include chickenpox (commonly known as varicella) and shingles (herpes zoster).

Incubation period for VZV varies from 10 to 20 days with an average time of 2 weeks; the virus it’s very contagious, and the infection usually spreads by inhalation of infective droplets or contact with lesions [250].

Symptoms of varicella in children are usually mild, with fever and malaise as their cornerstone, while in adult’s symptomatology can be more invalidate. Moreover, a pruritic rash is typical of varicella, and involves the face, scalp, trunk and eventually the extremities, with a maculopapular eruption that changes during the evolution of the disease to become pustular and later form crusts. 

In case of VZV reactivation, the pathology develops as herpes zoster with a cutaneous rash very similar to varicella’s one and severe pain that could precede the insurgence of dermatologic lesions [251].

### 20.1. Pathogenesis

Primary infection is transmitted by inhalation of viral particles; the virus has a tropism for airway cells, then replicates and enters the bloodstream causing viremia and the typical skin lesions of varicella. After primary infection, the virus rests in latency in primary neurons of neuraxis and can be reactivated during the life, causing a secondary infection called herpes zoster or “shingles” with lesions that follow a dermatomal distribution [252].

### 20.2. Cardiac Clinical Findings

VZV is recognized to be a virus associated with cardiac tropism and related symptomatology. It was first described in 1953, its role in the development of viral myocarditis was later found to be a primary factor in the development of supraventricular and ventricular arrhythmias in paediatric patients; and even more recently, this virus has been identified as a risk factor for stroke, myocardial infarction, and heart failure [253,254].

The cause identified by most prominent study in this regard, published by Yang et al. [255], identified the cause of cardiac concerns during VZV infection to be associated with the migration of viral particles from neural cells to vascular cells of the brain and heart, thus inducing an inflammatory response that could end up with occlusion in the bloodstream, ischemia, and infarction, or an autoimmune response with the formations of auto-IgM/IgG antibodies directed against lupus anticoagulant and cardiolipin molecules [256]. 

Moreover, in case of secondary viral infection, diffusion from the root of dorsal ganglia could result in the development of vasculopathy, ischemia, infarction, and heart failure caused by the rupture of the internal elastic lamina, intimal hyperplasia, and a general reduction of smooth muscle cells in the medial layer [257,258].

### 20.3. Cardiac Histology

At histology, VZV could be detected in the adventitial layer of coronaries, and trans axonal spread of reactivated VZV to the arterial adventitia followed by a transmural spread of the virus [259].

Heart vessels infected by VZV usually present:

- Disrupted internal elastic lamina;
- Thickened intima, whose myofibroblasts usually produce Alpha-smooth muscle actin that could potentially contribute to the narrowing of vessels lumen and development of ischemia;
- Loss of vessel wall integrity [260].

Large amounts of neutrophils could be identified in the arterial adventitia and ventricular walls; some authors suggest that these neutrophils (that produce elastase and metalloproteinases) could play a role in cardiac remodelling following VZV infection when oxygen species are produced in response to infection and smooth muscle cell proliferation. Migration could be prominent, which induces apoptosis and loss of smooth vascular cells [261].

In conjunction with activated metalloproteinases (MMP) directly secreted by VZV-infected vascular cells, MMP, and elastases can lead to extracellular matrix breakdown, weakening of the vessel wall, and aneurysm formation [262,263].

### 20.4. Cardiac Immunohistochemistry and Immunofluorescence 

Following histological patterns of VZV infections that involve the heart, CD4+, CD8+ and CD68+ T cells were detected, in conjunction with macrophages and CD20+ B cells found at immunohistochemistry; those cellular elements were rather abundant in adventitial and intimal walls but not in the tunica media.

### 20.5. Genetic and Blood Markers

No specific genetic mutation has demonstrated to be associated with heart complications following VZV infections yet. Gene susceptibility include variations of *HLA-S* gene, *HCG4P5* (belonging to HLA) and *ABHD16A* a 21-exon gene which encodes for the main brain phosphatidylserine (PS) hydrolase [264,265].

High levels of IL-1, IL-6 and IL-17 can be found, while a positivity for anti-VZV IgM and IgG can also be detected [266].

## 21. HIV Related Cardiopathy 

Human immunodeficiency virus (HIV) is an RNA virus first isolated in 1983 at the Pasteur Institute, Paris, and at the National Cancer Institute of Bethesda, Maryland. Epidemiologists believe that HIV infection’s peak was reached in 1999, and, as of 2023, the number of infected individuals has reduced by approximately 20% since its highest figure. About 15 million people worldwide suffer from HIV infection, especially in lesser developed countries. In comparison, about 5 million people can get the necessary care, thus reducing the incidence of AIDS, the final stage evolution of HIV infection [267]. 

Two are the types of HIV known as of now: HIV-1, responsible for 80% of HIV infections and more diffused worldwide, representing by far the most common HIV etiologic agent in Third World countries and Western countries, and HIV-2, responsible for a good 20% of infections, and more diffused in Western Africa than anywhere else [268]. 

The first symptoms appear 2–3 weeks after infection, with a flu-like pattern comprising fever, chills, rash, night sweats, muscle aches, sore throat, fatigue, swollen lymph nodes, and mouth ulcers. It is widely known that HIV patients are at higher risk of developing heart conditions than the general population, with a risk of cardiac involvement higher for HIV-1 infections than HIV-2 infections; of all mortality related to HIV infection, only 6.5% is considered related to cardiovascular disease. Besides, cases of HIV-related cardiomyopathy have risen in incidence after HIV has become a chronic disease. Heart conditions include pericardial and myocardial disease, heart failure, and pulmonary hypertension. 

Most recent diagnostic methodologies include the research of HIV antigens (especially the p24 antigen), anti-HIV antibodies, or HIV-RNA in blood samples [269].

### 21.1. Pathogenesis 

After viral transmission, the evolution of the infection occurs in three main phases: acute phase (primary infection), clinical latency, and symptomatic phase. 

Most new infections are linked to viral variants that use CCR-5 as a receptor. At the same time, CXCR-4 tropic viruses generally appear in the last stages of infection and have been connected to increased pathogenicity and speed progression of the disease [270,271].

The acute phase (primary infection) refers to the period between infection and its detection through an antibody test. Literature findings report 47.4–129.8 days (median 88.6d.) for the primary infection, which is further divided into five different stages, whose duration significantly differ individually, taking for granted that Stage V is the longest-lasting one (approximately 70 days):

– Stage I—Only viral RNA can be found;
– Stage II—Positivity for p24 antigen;
– Stage III—Development of Anti-HIV—IgM;
– Stage IV—Undetermined Western Blot analysis;
– Stage V—Reactive Western Blot.

Symptomatology, especially flu-like symptoms, develops between 4 days and weeks after first exposition to the viral agent and lasts 1 to 3 weeks on average [272]. 

Clinical latency is characterised by more significant destruction of T-lymphocytes CD4+ cells. This phase usually lasts for years until the eventual development of Acquired immune deficiency syndrome (AIDS). The frequent lack of symptoms usually hides the viral replication, which is still on course during the latency period; patients with T CD4+ levels > 500 cells/μL are usually asymptomatic (only dermatologic signs such as dermatitis, psoriasis, etc.) with exclusive signs of modest lymphadenopathy. 

Symptomatic phase: usually develops within ten years after infection. During this stage, a significant increase in viral load could be found, with a concomitant reduction of the T CD+ population beneath 350 cells/μL and a reduction in antiviral response by T CD8+ lymphocytes. In this phase, we could assist in the recrudescence of previous symptomatology, development of infections, and malignant neoplastic diseases [273]. 

### 21.2. Cardiac Clinical Findings

HIV disease is among the most critical causes of dilated cardiomyopathy, with a prevalence of 3.6% among subgroups of patients affected by this disease; the incidence of cardiomyopathy increases proportionally with the age of the patient himself [274,275].

One of the most studied causes of dilated cardiomyopathy is myocarditis; this statement is supported by the fact that dendritic cells play a significant role in the interaction between HIV and the myocyte and in the activation of multifunctional cytokines (such as TNF-α, interleukin-1, 6 and 10) that share a function in cardiac tissue damage [276,277].

HIV patients showed an accelerated ratio of coronary artery disease and hypertension; HIV patients can also experiment with developing inflammatory vascular diseases like polyarteritis nodosa, Henoch-Schoenlein purpura, and vasculitis induced by drug-induced hypersensitivity [278].

### 21.3. Cardiac Histology

The lack of lymphocytic infiltrate is a general feature of the pathology. Besides, detecting a wide macrophagic and neutrophilic infiltrate of all cardiac structures is possible, involving more the left and right ventricular walls [279].

### 21.4. Cardiac Immunohistochemistry and Immunofluorescence 

In immunohistochemistry, not only regarding heart tissue but also others, it is remarkable the positivity for cytokine factors such as IL-1, IL-6, IL-8, and IL-12, while there is a decrease in CD4 T cell presence.

The major antigen detectable for HIV infection is p24, whose presence can be highlighted not only in the serum of the patients but also in immunohistochemistry and immunofluorescence [280]. Antibodies, especially HIV-1 and HIV-2 IgM, can also be detected as signs of infection.

### 21.5. Genetic and Blood Markers

Genetic factors involved in the occurrence of the disease are linked to the HLA system; among them relevance (as reported by current literature) of *HLA-B57*, *HLA-B58*, *HLA-B27*, *HLABw4*, *HLA-A11* and *CCR5* [281,282].

## 22. Discussion

Autopsy diagnoses typically rely on observations of macroscopic, microscopic, and histological features. However, genetic, and molecular elements have gained increasing significance in contemporary practice. These instruments can identify previously undetectable markers or are prohibitively expensive to analyse. The integration of genetic and molecular techniques has expanded the diagnostic potential of autopsies, enabled the identification of subtle molecular abnormalities, and provided valuable insights into the underlying pathogenesis of diseases [283].

Diagnosing vasculitis, connective tissue disorders, and post-viral autoimmune diseases poses considerable challenges for forensic pathologists. However, advancements in medical technologies have played a pivotal role in facilitating the detection of relevant immuno-histochemical, genetic, and haematological markers previously unfamiliar or prone to misdiagnosis. Furthermore, the integration of artificial intelligence is emerging as a promising tool that can serve as a valuable resource for pathologists, providing essential support and aiding in the diagnostic process. As these technologies continue to evolve, they hold great potential to enhance the accuracy and efficiency of diagnosing autoimmune diseases in forensic pathology [284,285,286].

Immunohistochemistry has significantly improved over the past few decades and has become an invaluable technique for studying tissue pathologies. It enables the simultaneous detection of multiple markers on a single tissue section, allowing for a comprehensive analysis of cell composition, cellular function, and intercellular interactions [287,288].

Technological progress in immunohistochemistry has introduced multiplex assays and mutation-specific markers, opening new avenues for identifying novel targets for autoantibodies and cluster differentiation molecules in studying heart involvement in autoimmune diseases [289,290,291]. Immunohistochemistry has proven essential when it comes to vasculitis, connective tissue disorders, and post-viral autoimmune diseases. While CD3 positivity is common in most vasculitis diseases, it may not be immediately apparent in connective, granulomatous, and post-viral diseases. 

Researchers have identified specific factors detectable in tissue samples that can aid in diagnosing cardiomyopathies and immune-based hematologic diseases. Examples include Eosinophil protein X for Churg-Strauss syndrome (CSS), E-selectin for lupus erythematosus syndrome (LES), and PAD1, 2, 3, 4 for rheumatoid arthritis (RA). Advances in genotyping technologies and the analysis of large patient cohorts have allowed scientists to discover new genetic factors involved in human autoimmunity patterns. These factors play a role in differentiating pathologies with similar clinical and histologic manifestations but possess distinct genetic patterns [292,293]. Rheumatoid arthritis, for instance, exhibits complex HLA-related genetics that differentiates it from other connective tissue diseases, such as systemic sclerosis. Similar distinctions can be observed in post-viral diseases associated with autoimmune syndromes, like SARS-CoV-2, HSV, VZV, HIV, EBV, and CVB3, where specific genes confer susceptibility to viral infection and increase the likelihood of developing autoimmune pathologies. Currently, post-mortem genetic tests still need to be of more utility due to the requirement of complete genetic sequencing, which is prohibitively expensive for screening cases of undiagnosed autoimmune cardiac disease. 

However, they may prove valuable in post-mortem cases with high clinical suspicion. Indeed, these tests can provide genetic counselling opportunities for living family members in the event of confirmed genetic positivity, like practices followed for other medical conditions [294,295]. Genetic testing should be utilised selectively, particularly in cases where other findings are unavailable, considering the specific needs and availability of resources. Blood markers also play a significant role in diagnosing autoimmune cardiomyopathies during life and post-mortem examinations [296]. 

Useful blood biomarkers can aid in tracing autoimmune and hematologic diseases during autopsy. Systemic autoimmune diseases often involve abnormal activation of immune cells, leading to the secretion of inflammatory cytokines and chemokines. In diseases like Kawasaki disease, blood markers such as tumour necrosis factor α (TNFα), interferon-γ (IFN-γ), interleukins (e.g., IL-6), and monocyte chemoattractant protein (MCP)-1 contribute to the development of the vasculitis process [297,298]. Autoantibodies detected in blood samples are particularly important in autoimmune syndromes, including Churg-Strauss syndrome (increased levels of Eotaxin 1, 2, 3), Sjogren syndrome (anti-Ro/SSA, anti-La/SSB antibodies), Takayasu arteritis (factor Petraxin-3), and systemic lupus erythematosus (anti-cardiolipin and anti-paraoxonase L antibodies). Some autoimmune heart diseases may not exhibit documented increases in specific cytokines, except for Takayasu arteritis, CSS, and rheumatoid arthritis. However, antibody detection remains crucial in suspected viral-induced diseases (e.g., EBV VCA IgM-IgG, EBV EA IgM) [299]. Looking ahead, promising technologies such as the molecular analysis of micro-RNAs (miRNAs) are being investigated for potential use in post-mortem diagnosis of these complex diseases. These advancements can potentially enhance our understanding and detection of elusive autoimmune diseases [298,299,300,301].

## 23. Conclusions

Autoimmune heart disease is a common complication associated with various vascular and non-vascular diseases. However, histological findings can be intricate and often overlap, especially when there is a lack of sufficient medical history. On the other hand, the abundance of information available can also lead to confusion for pathologists in determining the most relevant and useful findings.

Currently, there are no standardised autopsy protocols or techniques specifically designed for suspected autoimmune cardiac pathologies. The various clinical manifestations of these conditions, resulting from variable organ involvement, present significant challenges. For example, cardiac diseases related to valve pathology can range from asymptomatic cases with minimal organ damage, particularly in young individuals, to severe congestive cardiopulmonary insufficiency in advanced age due to significant valve impairment. Consequently, forensic pathologists should rely on the available medical history to select appropriate autopsy techniques, prioritising the sampling of the most affected cardiac areas for subsequent histological examination. Notably, this study emphasises the recurrent involvement of specific cardiac regions in the same pathological context. We recommend performing standard cardiac sampling and additionally incorporating more specific sampling, particularly from pericardial and valvular sites. 

Blood samples can be collected during or after histological investigations to study markers such as cytokines or antibodies. Subsequently, the pathologist can determine the specific markers to be quantified in the laboratory, considering the clinical and autopsy suspicion. These complementary tools are intended to contribute to the overall diagnostic process.

Table 13 serves as a comprehensive summary guide, presenting an overview of the primary cardiac lesions observed in vasculitis, connective tissue disorders, granulomatous diseases, and post-viral conditions. Its purpose is to provide a comprehensive understanding of these pathologies by encompassing not only the associated clinical findings but also highlighting the pertinent histological, immunohistochemical, and genetic factors that contribute to their development. This resource aims to support pathologists in their diagnostic process.

The following table should serve as a summarizing guide to the main cardiac lesions occurring in vasculitis, connective, granulomatous and post-viral diseases, with a particular focus not only to the clinical findings of such pathologies, but also to histology, immunohistochemical and genetic factors that concur in the determinism of them.

## Figures and Tables

**Table 1:** Table shows the classification of autoimmune diseases with cardiac involvement.

Connective Tissue Diseases	Vasculitis	Granulomatous Disease	Autoinflammatory Disease	Post-Infective
– Systemic lupus erythematosus (SLE); – Rheumatoid arthritis; – Systemic sclerosis; – Mixed connective tissue disease; – Sjogren syndrome; – Polymyositis and dermatomyositis.	Small vessels	– Sarcoidosis.	– Familiar mediterranean fever; – TNF receptor-1 associated periodic syndrome (TRAPS)	Bacterial
	– Eosinophilic granulomatosis with polyangiitis (Churg Strauss); – Granulomatosis with polyangiitis (Wegener’s granulomatosis).			Rheumatoid fever.
	Medium-sized vessels			Viral
	– Polyarteritis nodosa; – Kawasaki disease.			SARS-CoV-2.
	Large vessels			
	– Takayasu arteritis; – Giant cell arteritis.			

**Table 2:** Clinical findings in CSS.

Clinical Findings	Comment
Asthma	History of wheezing or diffuse high-pitched expiratory rhonchi
Eosinophilia	Eosinophilia > 10% on differential white blood cell count
Mono or polyneuropathy	Development of mononeuropathy, multiple mononeuropathy, or polyneuropathy attributable to systemic vasculitis
Paranasal sinus abnormality	History of acute or chronic paranasal sinus pain or tenderness or radiographic opacification of paranasal sinuses
Extravascular eosinophils	Extravascular areas

**Table 3:** Cardiac clinical findings in CSS.

Cardiac Clinical Findings	
Pathological forms	Coronary artery disease Myocardial infarction Congestive heart failure Pericarditis Myocarditis Massive pericardial effusion Sever valvular insufficiency Cardiac tamponade Arrythmias
Symptoms/Signs	Chest pain Palpitations Cardiogenic shock Sudden death
Evidence on Imaging	Focal fibrosis Wall motion disturbances Oedema Increased left ventricular cavity dimensions Abnormalities in small vassals as necrotizing vasculitis or thrombi in ventricular cavities

**Table 4:** Cardiac clinical findings in TA.

Cardiac Clinical Findings	
Pathological forms	Coronary artery disease Heart valves disease. Aortic insufficiency (62.7%) and mitral insufficiency (41%) Myocarditis Myocardial infarction

**Table 5:** Clinical features PAN.

System	Clinical Features	Signs/Symptoms
Constitutional		Fever Weight loss
Renal	Ischemia Infarction Nephropathy	Hypertension Hematuria Albuminuria
Skin	Ulcers Nodules Purpura Livedo reticularis Edema	
Gastrointestinal tract	Bowel infarction	Abdominal pain Melena
Ocular	Retinal hemorrhage Optic ischemia	Visual impairment
Nervous system	Mononeuritis multiplex	Sensory symptoms preceding motor deficits

**Table 6:** Cardiac clinical findings PAN.

Structure	Clinical features	Frequency
Coronary artery	Aneurysmal degeneration Thrombocclusion phenomena	30%
Myocardium	Myocardial infarction Myocarditis Cardiomegaly	3%
Conduction system	Arrhythmia	<1%

**Table 7:** Table shows the ICBD-point score system.

Sign/Symptom	Points
Ocular lesions	2
Genital aphthosis	2
Skin lesions	2
Neurological manifestations	1
Vascular manifestations	1
Positive pathergy test ^1^	1

^1^ Pathergy test is optional.

**Table 8:** Cardiac clinical findings in BD.

Structure	Clinical Manifestations
Coronary vessels	Vasculitis: stenosis, thrombus, aneurysm
Endocardium	Endocarditis Endomyocardial fibrosis Valve diseases (aortic valve > mitral valve) Intracardiac thrombus
Myocardium	Unstable angina Myocardial infarction Cardiomyopathy
Pericardium	Pericarditis Hemorrhagic pericardial tamponade Constrictive pericarditis Pericardial effusion

**Table 9:** Table shows five main features for the diagnosis of complete KD.

Criteria	Clinical Features
Oral and lip lesions	Erythema and cracking of lips “Strawberry tongue”
Conjunctivitis	Bilateral bulbar non exudative conjunctival injection
Polymorphous rash	Maculopapular diffuse erythroderma or erythema multiforme-like. Actue pase: erythema and edema of the hands and feet.
Extremity changes	Subacute pase: periungual desquamation.
Lymphadenopathy	Acute, non-suppurative, cervical lymphadenopathy, tipically unilateral.

**Table 10:** Histological features of vascular.

Stage	Histological Features
Stage 1 Degeneration of endothelial cells	Hyperplastic and proliferative endothelial cells. Degeneration and desquamation endothelial cells. Fibrin mass including platelets and inflammatory cells form a minute parietal along the degenerated and desquamated endotheliial cells.
Stage 2 Edemea and degeneration of the media	Inflammatory infiltration in the edematous, thickened intima. Vacuolization of the muscle cells. Edema of the media.
Stage 3 Necrotizing panarteritis	All layers of the arterial wall extensively destroyed with numerous inflammatory infiltrations. Desquamation, degeneration and necrosis of the endothelial and muscle cells. Proliferation and swelling of collagen fibers.
Stage 4 Granulation formation	Granulation tissue in the intima and Media. Fibrinoid material along the luminal surface or in the subendothelial space.
Stage 5 Scar formation	Fibrous connective tissue in place of the intima and media. Proliferation of collagen and elastic fibers in the adventitia or perivascular area. Lumen stenotic or occluded.
Stage 6 Aneurysm formation	Dilatation of the lumen and thinning of the vascular wall (aneurysm formation). The lumen of aneurysm often completely occluded by a trhombus Undistinguishable three laminar structures of the arterial wall.

**Table 11:** Table shows diagnostic criteria of Rheumatic Fever. “CRP” means C-reactive protein, while “ESR” means Erythrocyte sedimentation rate.

	Low Risk Population	Moderate/High Risk Population
Definition	Annual incidence of ARF < 2 per 1,000,000 school aged children or all age prevalence of RHD < 1 per 1000	Those not fulfilling criteria for low risk
Major manifestations		
Joint manifestation	Polyarthritis	Polyarthritis and/or polyarthralgia Monoarthritis
Carditis	Clinical and/or subclinical	Clinical and/or subclinical
	Chorea	Chorea
	Erythema marginatum	Erythema marginatum
	Subcutaneous nodules	Subcutaneous nodules
Minor manifestations		
Carditis	PR prolongation (age adjusted)	PR prolongation (age adjusted)
Arthralgia	Polyarthralgia	Monoarthralgia
Fever	>38.5 °C	>38 °C
Inflammatory markers	ESR > 60 mm in first hour and/or CRP > 3.0 mg/dL	ESR > 30 mm in first hour and/or CRP > 30 mg/dL
Supporting evidence of antecedent GAS infection: ➢ Increased or rising anti-streptolysin title or other streptococcal antibodies (anti DNAseB) ➢ Positive throat culture for GAS or positive rapid GAS carbohydrate antigen First episode of ARF: 2 major or 1 major plus 2 minors (in the presence of supporting evidence) Recurrence: 2 major or 1 major plus 2 minor or 2 minors (in the presence of supporting evidence)		

**Table 12:** Cardiac clinical findings COVID-19.

Structure	Clinical Features	Frequency
Myocardium	Left Ventricular Systolic Dysfunction Cardiogenic shock	35–67%
Coronary vessels	Vasculitis: from mild dilatation to the development a giant aneurysmatic dilatation	6–24%
Conduction system	Arrythmia ECG alterations	7–60%

**Table 13:** The table summarises the major heart diseases induced by autoimmune diseases. It involves three significant spheres: vasculitis pathologies, connective tissue pathologies, and post-viral pathologies. Only histological and immunohistochemical examinations performed on cardiac tissue have been considered. The following acronyms were used: MISC means “multisystemic inflammatory syndrome-children”, XIAP means “X-linked inhibitor of apoptosis”, CYBB means “cytochrome b-245, beta subunit”, RF means rheumatoid factor; p-ANCA means “Perinuclear antineutrophil cytoplasmic antibodies”, ANA means “Antinucleous Antibodies”, TNFRI means Tumor Necrosis Factor receptor, anti-CCP means “Anti-cyclic citrullination peptide”, ACPA means “anticitrullinated peptide antibodies”, IL-6 means “Interleukin 6”, HDL means “high-density lipoprotein”, IRF means “interferon regulatory factor”, TNFAIP3 means TNF-α-induced protein 3; TNFSF4 means “TNF ligand superfamily member 4”, PTPN22 means “Protein tyrosine phosphatase, non-receptor type 22”; BANK1 means “B cell-specific scaffold protein with ankyrin 1”, ALT means “alanine aminotransferase”, AST means “aspartate aminotransferase”, ESR means “erythrocyte sedimentation rate”, ASO means “Antistreptolysin”, CD24v means “CD24 nucleotide alanina substitution with valina”; CK means “Creatin kinase”, CAM means “cell adhesion molecules”, TF1 means “Tumor suppression protein”, cTnT means “troponin C”, ITnT means “troponin I”. KIR means “Killer-Cells Immunoglobin like Receptor”; NKG2C means “Natural Killer G2C receptor”, TRAIL-R 3 means “Trail receptor-3”, CCL17/TARC means “CCL17/thymus and activation-regulated chemokine”.

Vasculitis					
Cardiopathy	Autoptic Findings	Histology	Immunohistochemistry	Genetic	Blood Markers
Churg-Strauss syndrome	*Endocardium* – Valve closure defect, particularly mitral valve – Thrombi in ventricular cavities *Myocardium* – Dyschromia area *Pericardium* – Fluid in pericardial cavity – Coronary vessels – Stenosis – Ectasia	Eosinophilic infiltration, Necrotizing small vessel vasculitis, Perivascular neutrophilic infiltrates, Lymphocytes infiltration, Endomyocardial fibrosis	P-ANCA+/− CD3 + CD68 + CD83 + ECP Eosinophil protein-X Eotaxin-3	HLA-DRB4 DNAM1s	ANCA+ ANCA− IL-2 - IL-4 - IL-5 - IL-13 - IL-14 - INF-a - INF-g - CK - Troponin I - Eotaxin 1 - Eotaxin 2 - Eotaxin 3 - CCL17/TARC - IgE - IgG -
Takayasu arteritis	*Endocardium* – Valve closure defect, in particularly aortic and mitral valves – Fibrosis and calcification of the aortic valve *Myocardium* – Dyschromia area – Increased of the heart chambers *Coronary vessels* – Stenosis – Aneurysmal dilatation	Lymphoplasmacytic infiltration, Myocitolisis, Myocardial hypertrophy.	CD3 + S-100 + CD15 +	HLA-B*52 IL-12B FCGR2A/3A FCγR2A/3 IL12B IL6 RPS9/LILRB3 Intergenic locus on chromosome 21q22	AACEA+ 86% AACEA− 9% IL-6 - IL-8 - IL-18 - Petraxin 3 - Serum amyloid A - HLA E -
Polyarteritis nodosa	*Myocardium* – Dyschromia area *Coronary vessels* – Stenosis – Aneurysmal dilatation	Localized necrotizing arteritis, Mixed inflammatory infiltrate.	TLR-4, CD3 + CD4 + CD22 +	MEFV DADA2	p-ANCA – ASO+
Benhcet’s disease	*Endocardium* – Thrombi in ventricular cavities *Myocardium* – Dyschromia area *Pericardium* – Fluid in pericardial cavity – Fibrous thickening *Coronary vessels* – Stenosis – Aneurysmal dilatation	Neutrophils infiltration, Leucocytoclastic formations.	MPO + CD3+ CD138+	Unspecific HLA involvement.	AECA anti CTDP-1 ANA-, ANCA- Anti MPO antibodies
Kawasaki disease	*Myocardium* – Dyschromia area *Coronary arteries* – Ectasia/Aneurysmal dilatation	Neutrophilic infiltration (first phase), Lymphocytic, eosinophil infiltration (second phase), Myofibroblast infiltration (third phase).	IVIG FCGR1a FCGR3A CCR2 S100A9 S100A12 adrenomedullin FCGR2A S100A9 S100A12	ITPKC, CASP3, CD40, ORAI, ABCC4	CD4 CD8 PCR ESR ALT AST Albumin Na+ K+ HDL-cholesterol
Connective Tissue					
Cardiopathy	Autoptic Findings	Histology	Immunohistochemistry	Genetic	Blood markers
Systemic eritematous lupus	*Endocardium* – Valve disease: vegetations, fibrosis and calcification of the valve flaps *Pericardium* – Fibrous thickening	Mononuclear cells infiltration in perivascular and interstitial tissues.	MHR nLHR High-density lipoproteins E-selectin	CD24v	ANTI-p AB, AECA Antibodies against paraoxonase 1; Anti-cardiolipin antibodies
Rheumatoid artrithis	*Myocardium* – Dyschromia area	Cardiac hypertrophy activated monocytes infiltration, macrophages infiltration, and T lymphocytes infiltration, myocardial and endothelial apoptosis, interstitial fibrosis and fibrotic bands.	TNFRI, anti-CCP, Citrullination, PAD1 in cytoplasmatic granules, PAD 2 in leukocytes, PAD 3 and PAD 4 in cardiomyocites	HLA-DRB1 01 HLA-DRB1 04 PTPN22 DRB1 ACPA	TNF-, - TNFRII messenger RNA - CD4 lymphocites - CD28 lymphocites - Anti-CD4 Ab - Anti-CD28 Ab IL-12 -
Acute rheumatic fever (Rheumatic heart disease)	*Endocardium* – Valve closure defect, particularly mitral and aortic valve *Myocardium* – Dilatation of left atrium and left ventricle – Dilatation of right atrium and left ventricle	Lymphocytic infiltration, Aschoff’s nodules, histiocytic aggregates, myocyte degeneration, interstitial degeneration, interstitial mononuclear cell infiltration.		HLA and IGH regions but still unclear	-Anti-streptococcum A antibodies
Sistemic sclerosis	*Myocardium* – Dyschromia area – Thickening of the right ventricular wall and increased right ventricular cavity dimension	Activated T-Lymphocytes infiltration, macrophage infiltration, fibrosis (8–32%).	CD3+ CD68+ HLA II + SM-actin +	IRF4 IRF55 TNFAIP3 TNFSF4 PTPN22, BANK1, IL-21 gene	ANA + (95%) IL-6 - IL-12 - IL-23 - TNF-a -
Sjogren syndrome	*Endocardium* – Valve closure defect *Myocardium* – Dyschromia area	Leukocytoclastic infiltration, macrophage infiltration, fibroblasts.	CD45 + anti-SSA/Ro Siglec-1 in macrophage in cardiac septal region	HLA-DR3 Id3 deficiency	anti-Ro/SSA - anti-La/SSB - C3, C4 - anti-phospholipid antibody -, triglycerides -, HDL -, (IL)-1β -, IL-6 - IL-2 - INF-g
Polimiositis and Dermatomiositis	*Endocardium* – Valve closure defect – Thickening of the valve flaps *Myocardium* – Dyschromia area *Coronary vessels* – Stenosis – Aneurysmal dilatation	Active myocarditis, focal myocardial fibrosis, vasculitis, intimal proliferation, medial sclerosis of vessels, lymphocytic infiltration, Conduction system fibrosis, myocardial fibrosis	CD59+ anti-Ro	MHC polymorfism, DNA methylation, Histone modification	Anti-Rho, CK-MB - anti-Mi2 - anti-MDA5 - anti-NXP2 - anti-TIF1 - anti-SAE - CTnT ITnT
Granulomatous Inflammations					
Cardiopathy	Autoptic Findings	Histology	Immunohistochemistry	Genetic	Blood markers
Sarcoidosis	*Myocardium* – Various and rare structural alterations	Lymphocytes infiltration, fibrosis.	CD4+, CD8+ CD15+ CD20+ CD68 IL6− S100− Ki67−	HLA-DRB1*0301	RF - ANA -
Post-viral					
Cardiopathy	Autoptic Findings	Histology	Immunohistochemistry	Genetic	Blood markers
MIS-C post-COVID-19	*Myocardium* – Increased left ventricular cavity dimension *Coronary vessels* – Aneurysmal dilatation	Activated T-lymphocytes infiltration in myocardium, mononuclear infiltration fibrin microvascular thrombi, non-specific myocardial edema.	IL-1 +, IL-6 +, IL 17-1 +, CXCL-10.	SOCS1 haploinsufficiency, XIAP, CYBB	IgG against HKU1 Leucocytes -, Neutrophils -, PCR -,
Post-COVID-19 cardiopathy	*Myocardium* – Dyschromic areas *Coronary vessels* – Stenosis/occlusion	Activated T-lymphocytes infiltration in myocardium, mononuclear infiltration, fibrin microvascular thrombi, non-specific myocardial edema, Necrosis.	IL 6+, CAM +,	Unknown	CK-MB -, CK -, Myoglobin -, Troponin -, NT-proBNP -
Coxsackie virus B-3 myocarditis	*Myocardium* – Dilation of cardiac chambers	Lymphocytes (Th2, Th17) infiltration.	Unknown	Unknown	Unknown
Epstein Barr cardiomyopathy	*Endocardium* – Valve closure defect – Thrombi in ventricular cavity *Myocardium* – Dilation of cardiac chambers	Lymphocytic infiltrate.	IL-1 +, IL-6 +, IL-17 +.	unknown	EBV Vca IgM > 20 EBV Vca IgG > 20 EBV EA IgG > 10 Myocardial necrosis markers (Troponins)
Herpes simplex cardiomyopathy	*Myocardium* – Dilatation of cardiac chambers	Lymphocytic infiltrate.	IL-1 +, IL-6 +, CD3 +, CD 68 +.	KIR NKG2C CD16A CD32A	HSV-1, HSV-2 IgM+ - HSV-1, HSV-2 IgG+ - HHV6-HHV7 AB -----
Varicella zoster virus (VZV)	*Myocardium* – Dyschromia areas	Lymphocytic, macrophagic infiltrate in myocardium and conduction tissue.	IL-1+ IL-6+ IL-17+	HLA-S, HCG4P5, ABHD16A	VZV IgM+ - VZV IgG+ -
HIV related cardiomiopathy	*Myocardium* – Dilatation of cardiac chambers	Macrophagic and neutrophilic infiltrate, low lymphocytic levels.	IL-1+ IL-6+ IL-8+ IL-12+ CD-4−	HLA-B57, HLA-B58, HLA-B27, HLA-Bw4 HLA-A11 CCR5-32	P24 antigen - HIV1-HIV2 -IgM -

## Data Availability

The data that support the findings of this study can be found in the databases used, specifically PubMed, Science Direct Scopus, Google Scholar, and Excerpta Medica Database (EM-BASE).
